# Understanding the solubilization of Ca acetylide with a new computational model for ionic pairs†

**DOI:** 10.1039/d0sc04752j

**Published:** 2020-10-08

**Authors:** Mikhail V. Polynski, Mariia D. Sapova, Valentine P. Ananikov

**Affiliations:** Saint Petersburg State University Universitetsky Prospect 26 Saint Petersburg 198504 Russia polynskimikhail@gmail.com val@ioc.ac.ru; Zelinsky Institute of Organic Chemistry, Russian Academy of Sciences Leninsky Prospect 47 Moscow 119991 Russia

## Abstract

The unique reactivity of the acetylenic unit in DMSO gives rise to ubiquitous synthetic methods. We theoretically consider CaC_2_ solubility and protolysis in DMSO and formulate a strategy for CaC_2_ activation in solution-phase chemical transformations. For this, we use a new strategy for the modeling of ionic compounds in strongly coordinating solvents combining Born–Oppenheimer molecular dynamics with the DFTB3-D3(BJ) Hamiltonian and static DFT computations at the PBE0-D3(BJ)/pob-TZVP-gCP level. We modeled the thermodynamics of CaC_2_ protolysis under ambient conditions, taking into account its known heterogeneity and considering three polymorphs of CaC_2_. We give a theoretical basis for the existence of the elusive intermediate HC≡C–Ca–OH and show that CaC_2_ insolubility in DMSO is of thermodynamic nature. We confirm the unique role of water and specific properties of DMSO in CaC_2_ activation and explain how the activation is realized. The proposed strategy for the utilization of CaC_2_ in sustainable organic synthesis is outlined.

## Abstract 2

Constructing the carbon framework from a carbon-neutral source: a new computational model for ionic pairs in solution based on DFTB MD and DFT helps to propose a strategy for sustainable organic transformations with solid CaC_2_.

## Graphical abstract

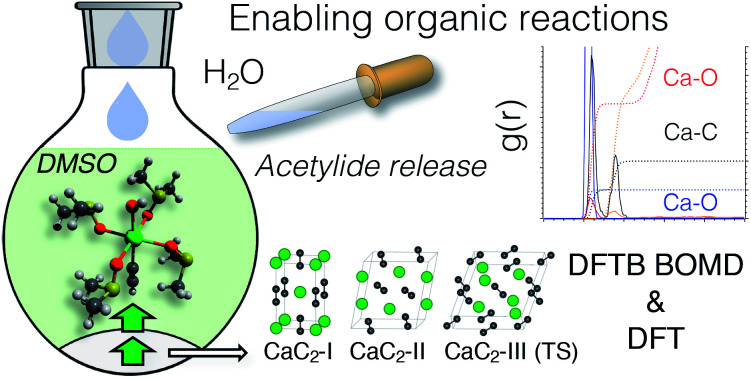

## Introduction

The construction of the carbon framework is one of the main goals of organic synthesis, and can be achieved using simple building blocks such as HC≡CH or, better, HC≡C^−^ and even C≡C^2−^. To obtain the latter two, one needs to use an acetylide source. Many metal acetylides are known, including acetylides of practically all classes of metals,^1–10^ and bi-metallic acetylides.^11–13^

Among metal acetylides, widely produced CaC_2_ now appears to be the most versatile choice for the synthesis of organic substances, including those that are biologically active,^14–24^ as well as monomers.^25–27^ Moreover, CaC_2_ is envisioned to become the feedstock for the sustainable, carbon-neutral chemical industry.^16,21,28^ It is also considered valuable or promising in the synthesis of nanostructured materials,^29,30^ agriculture,^31–34^ and metallurgy (alloy making, see Section 2.3.8 in ref. 28). However, CaC_2_ is insoluble in organic solvents, which hampers its reactivity in the liquid phase.^19,21,23,35,36^

Preformed acetylides and acetylide intermediates play a key role in organic synthesis. Copper-catalyzed azide–alkyne cycloaddition (CuAAC)^37,38^ is a widely used reaction, in which the main intermediate is the unstable Cu acetylide having a Cu–C≡C–R moiety. The use of a preformed acetylide makes the reaction significantly more facile.^39^ Other organometallic acetylides such as those of Au,^40^ Bi,^39^ and Pt^41^ also undergo dipolar cycloaddition to azides; the corresponding AAC reaction products are potent precursors to a wide range of substituted heterocyclic compounds.^39^

Activation of HC≡CH or RC≡CH *via* acetylide formation is necessary in CuAAC^42–45^ and other^46^ reactions. It was proposed that “any *s*-acetylide that can effectively recruit a p-bound copper atom will undergo annulation with a compatible dipolar partner.”^43^ Ca acetylides undergo dipolar cycloadditions as well.^14,47–49^

Acetylide species, like HC≡C–Ca–OH, are often assumed to be intermediates in solution-phase organic reactions with CaC_2_ that is insoluble by itself;^19,23,50^ however, it is hard to detect these species in the liquid phase. Detection of soluble alkaline acetylides was reported under extremely basic conditions.^35,51^ Ca acetylide was experimentally detected with Fourier transform infrared spectroscopy in solid CaC_2_ in a KBr matrix when subjected to trace amounts of H_2_O.^52^ Acetylene chemistry in dimethyl sulfoxide (DMSO) under basic and super-basic conditions is a valuable and indispensable tool of modern organic chemistry.^46,53–56^ Greater potential of practically valuable synthesis with CaC_2_ can be realized through understanding the unique performance of DMSO solutions.

Quantum chemical modeling of Ca acetylides in DMSO, reported below, required innovative consideration of ionic pairs in solution that have strong solute–solvent interactions. To obtain consistent models, we combined conformational sampling by molecular dynamics (MD) with the density-functional tight-binding (DFTB) Hamiltonian followed by DFT post-treatment of the conformations and free energy computations. Conformational sampling with fast semi-empirical methods has seen tremendous development recently.^57–60^ Combining them with DFT post-treatment allows, *e.g.*, estimation of realistic IR spectra in solution^61^ and reliable exhaustive conformational sampling of organic macrocycles.^62^

Given the importance of CaC_2_ as a sustainable carbon source for organic synthesis and Ca acetylides as potent intermediates, we performed this computational study. Obtaining active acetylide intermediates is key to new solution-phase organic reactions with solid CaC_2_. As the main result, we propose a strategy for the development of new sustainable solution-phase transformations based on the utilization of CaC_2_.

## Results and discussion

### Thermodynamic model

**Fig. 1:**
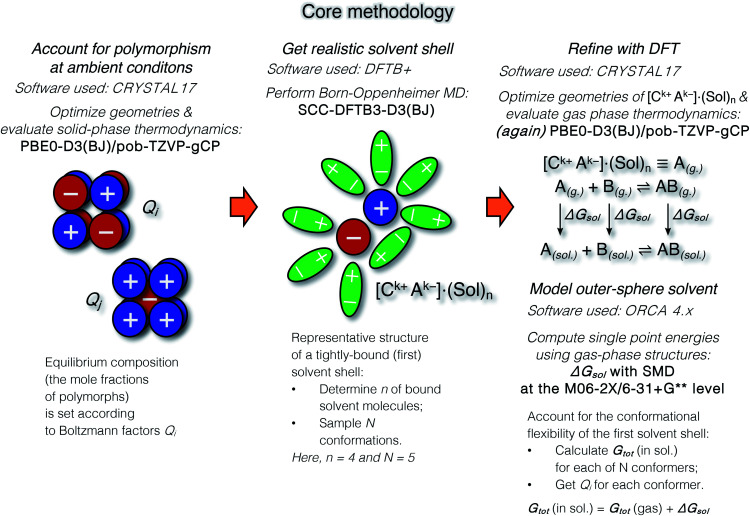
The core methodology and software used to perform the calculations outlined in Fig. 2.

The suggested strategy for modeling the dissolution of ionic solids, possibly including partial solvolysis and (or) solvent coordination, combines DFTB molecular dynamics and static DFT computations. Fig. 1 depicts a schematic description of the methodology. All parameters are listed in the Computational details section below and in the ESI.† By using the suggested methodology, it is possible to obtain dissolution free energies (following all three stages in Fig. 1), as well as to model chemical transformations of ionic pairs in polar coordinating solutions (performing computations in the last two stages).

**Fig. 2:**
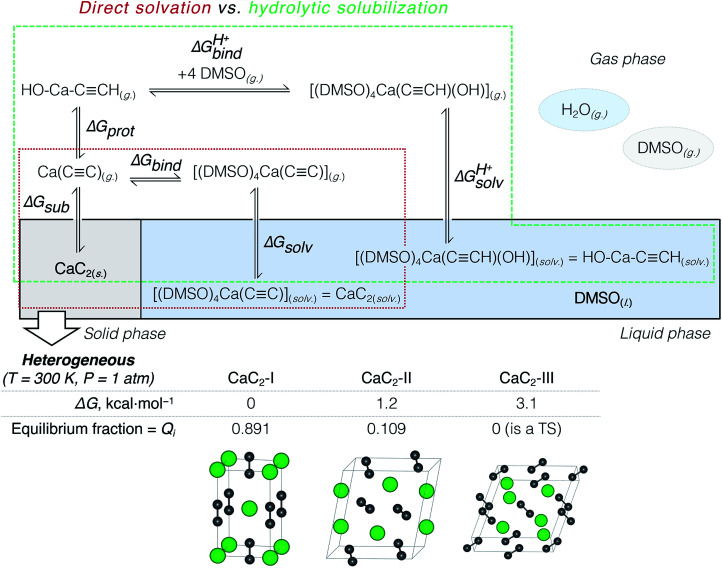
Thermodynamic model: direct CaC_2_ solvation in DMSO *vs.* partial hydrolysis in the water/DMSO system (hydrolytic solubilization). The heterogeneity of CaC_2_ was accounted for by considering two polymorphs with fractions equal to their Boltzmann weights (*Q*_*i*_).

According to previous studies, Ca acetylide can undergo organic transformations in DMSO^19,20,23,48,50^ and dimethylformamide^63^ solutions upon the addition of water. That is why the consideration of partial hydrolysis is essential. We compare direct CaC_2_ solvation and solubilization involving partial hydrolysis by considering the elementary steps depicted in Fig. 2: the consideration starts from solid CaC_2_ and proceeds in the clockwise direction to solvated species. Note that the states of the intermediates (solid, gas, solvated) are explicitly defined in Fig. 2. We use an analog of the Born–Haber cycle, and model the solvation and hydrolysis as the sequence of hypothetical sublimation (Δ*G*_sub_), reactions in the gas phase (Δ*G*_bind_, Δ*G*_prot_, Δ*G*^prot^_bind_), and the subsequent solvation (Δ*G*_solv_, Δ*G*^prot^_solv_).

We calculated Boltzmann weights for the stable CaC_2_ polymorphs, isomers and conformers:
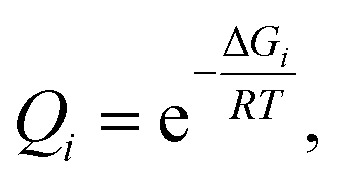
where Δ*G*_*i*_ is the relative Gibbs energy of the *i*-th isomer, conformer, or polymorph. *Q*_*i*_ values obtained in this way were used to compute the average free energies of species in solution.

### Solid Ca carbide and its sublimation

CaC_2_ is reported to be a mixture of three polymorphs CaC_2_-I, CaC_2_-II, and CaC_2_-III under ambient conditions.^64–66^ Because of the inconsistencies in previous studies,^64,65^ we present a more detailed discussion of our findings on the relative stability of CaC_2_ polymorphs in Section S2.†

We compared the stability (relative Δ*G*) of these three phases at 200, 300, and 400 K and found that the well-known tetragonal CaC_2_-I form is the most stable. The equilibrium distribution of stable CaC_2_ phases under standard conditions was estimated by computing Boltzmann weights according to the calculated Δ*G* values (see Fig. 2 and the ESI† for details).

According to the harmonic vibrational mode analysis at the PBE0-D3(BJ)/pob-TZVP-gCP level (see Section S2† for details), CaC_2_-III has an imaginary frequency at *Γ* point, so we excluded it from the set of allowed thermodynamic states for the sake of model consistency. Excluding CaC_2_-III from the calculation of Δ*G*_sub_ in Fig. 2 resulted in a negligible correction of less than 0.1 kcal mol^−1^ due to its relatively high free energy. In contrast, we did not observe any imaginary modes at the chosen level in the cases of CaC_2_-I and CaC_2_-II. The relative and absolute stability of CaC_2_ polymorphs remains unclear under theoretical considerations with computational methods (see the discussion of the relevant literature in Section S2.1†).

It was hypothesized that anharmonic effects may affect the stability of CaC_2_ phases.^65^ We believe that further investigation of potential energy surfaces of CaC_2_ polymorphs may be worthwhile, ideally, with Born–Oppenheimer MD (BOMD), to elucidate possible anharmonicity of atomic vibrations. As long as the proposed methodology (Fig. 1) is modular, any refinements of Δ*G* values can easily be incorporated.

The first elementary reaction to consider is the sublimation of CaC_2_ (Δ*G*_sub_). Computed at the PBE0-D3(BJ)/pob-TZVP level, the free energy of sublimation only slightly varied for the stable polymorphs: from 184.5 (CaC_2_-II) to 185.8 kcal mol^−1^ (CaC_2_-I). After Boltzmann averaging over stable CaC_2_ polymorphs we obtained 185.6 kcal mol^−1^ for the two-phase acetylide.

### Ionic pairs in realistic solvent

DMSO solvates cations very efficiently, even more strongly than water.^67,68^ Exergonicity of cation solvation in DMSO can be attributed to the formation of strong cation–oxygen bonds in the coordination shell. Using implicit solvent models and neglecting direct Ca–O-bonding when modeling the solvation of CaC_2_ and HC≡C–Ca–OH ionic pairs in DMSO leads to inconsistent results, as demonstrated in Section S3.†

We used Born–Oppenheimer molecular dynamics with the dispersion-corrected DFTB3-D3(BJ) Hamiltonian to determine the solvation shell of ionic pairs HC≡C–Ca–OH and [Ca^2+^][C_2_^2−^] in DMSO. First, we performed 10 ps-long isobaric-isothermal MD runs with the Berendsen thermostat and barostat to equilibrate the systems. Plots depicting the relaxation of the thermodynamic parameters *V*, *P*, and *T*, as well as of the sum *U* + *PV* + *TS*_elec_, are given in Section S5.†

**Fig. 3:**
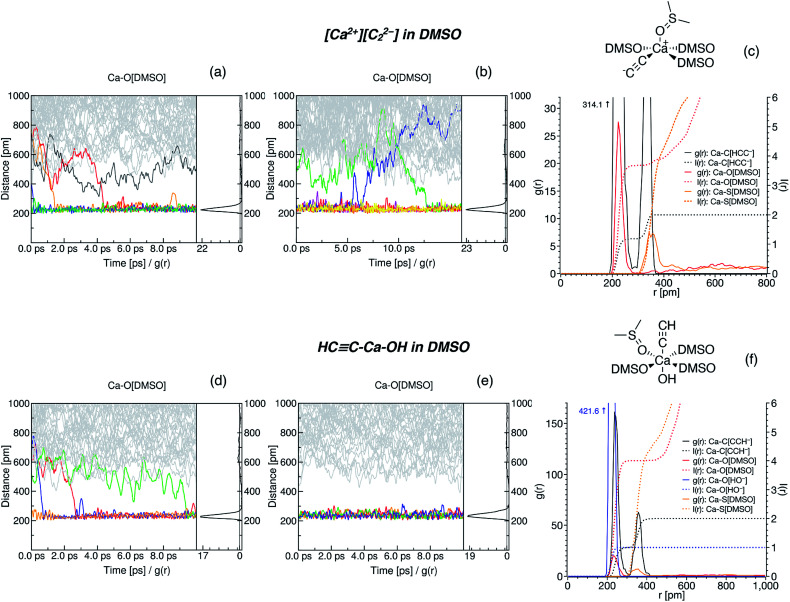
Radial distribution functions in the [Ca^2+^][C_2_^2−^]/DMSO (a–c) and HC≡C–Ca–OH/DMSO (d–f) systems. Time evolution of the interatomic distances is shown in (a), (b), (d) and (e); (a) and (d) depict the evolution in the equilibration runs, (b) corresponds to the simulated annealing run, and (d) corresponds to the sampling NPT run. Equilibrated structures of the Ca^2+^ solvation sphere are shown in (c) and (f). In (c), the RDFs in the last 5 ps of the run (b) are shown (cooled to 300 K after the annealing); in (f), the RDFs in the whole run (e) are depicted. Integral functions I(r) show the time-averaged CN of Ca^2+^. For clarity, the structural formula is shown only for one solvent molecule. Other solvent molecules are abbreviated as DMSO; they are also coordinated *via* O atoms.

Time evolution of the radial distribution functions (RDFs) demonstrates the equilibration of Ca^2+^ coordination number (CN, see Fig. 3 and Section S5†). Four DMSO molecules rapidly coordinate Ca^2+^ in the system with [Ca^2+^][C_2_^2−^]. In the system with HC≡C–Ca–OH, in contrast, the fourth DMSO molecule bonded to Ca^2+^ only in the last picosecond of the equilibration run (Fig. 3d).

Next, we subjected the HC≡C–Ca–OH system to another 10 ps NPT run, now using the Nosé–Hoover chain thermostat and Berendsen barostat, to sample the configuration space (Fig. 3e). The model of CaC_2_ in DMSO was subjected to simulated annealing (NVT ensemble, Nosé–Hoover chain thermostat) by gradually heating the system to 600 K for 3 ps, preserving the temperature for 5 ps, gradually cooling the system for 3 ps, and then keeping the temperature at 300 K for another 5 ps.

In the sampling run, we observed no additional binding of DMSO molecules in both systems (Fig. 3e); analogously, no additional DMSO molecules were bound as a result of the annealing (Fig. 3b). The resulting CNs are obtained from the integrals of the RDFs (Fig. 3c and f). Evidently, Ca^2+^ is six-coordinated in the HCC–Ca–OH system, which agrees with the experimentally observed CN of six for this cation in DMSO solutions.^69^ One may consider C_2_^2−^ as a κ^2^- or, equally, η^2^-ligand. In dynamics at 300 K, however, C_2_^2−^ mostly resides in the singly coordinated mode, which is why the second peak is present on the corresponding RDF at ∼340 pm (Fig. 3c and Section S6†). Since such behavior of C_2_^2−^ was unexpected, we performed simulated annealing of [Ca^2+^][C_2_^2−^] in DMSO instead of an NPT run to check if the solvent shell would equilibrate to the same CN after the annealing and no more DMSO molecules would bind to Ca^2+^. We suppose that C_2_^2−^ strongly electrostatically repels O-centers in DMSO, so only 4 DMSO molecules could bind to Ca^2+^ under the selected computational protocol.

We performed Boltzmann averaging over the ensembles of solvated [Ca^2+^][C_2_^2−^] and HC≡C–Ca–OH to obtain a conformationally sampled structure of Ca^2+^ solvation shell. For each system, we took 5 snapshots at distant trajectory points and cut Ca^2+^ with its first solvation shell representing a new model system for step 3 in Fig. 1, right (see Section S1.6† for details). Also, for both systems, we manually constructed conformations of the solvation shell by symmetrically placing 4 DMSO molecules in the equatorial plane of [(DMSO)_4_Ca(C≡CH)(OH)] and in the base of the tetragonal pyramidal [(DMSO)_4_Ca(C≡C)]. The latter artificial conformations were included as a stress test of the presented methodology. As shown below, these artificial conformations are negligible contributors to the pool of conformers. Geometries of all snapshot conformations obtained in this way were optimized at the PBE0-D3(BJ)/pob-TZVP-gCP level.

**Fig. 4:**
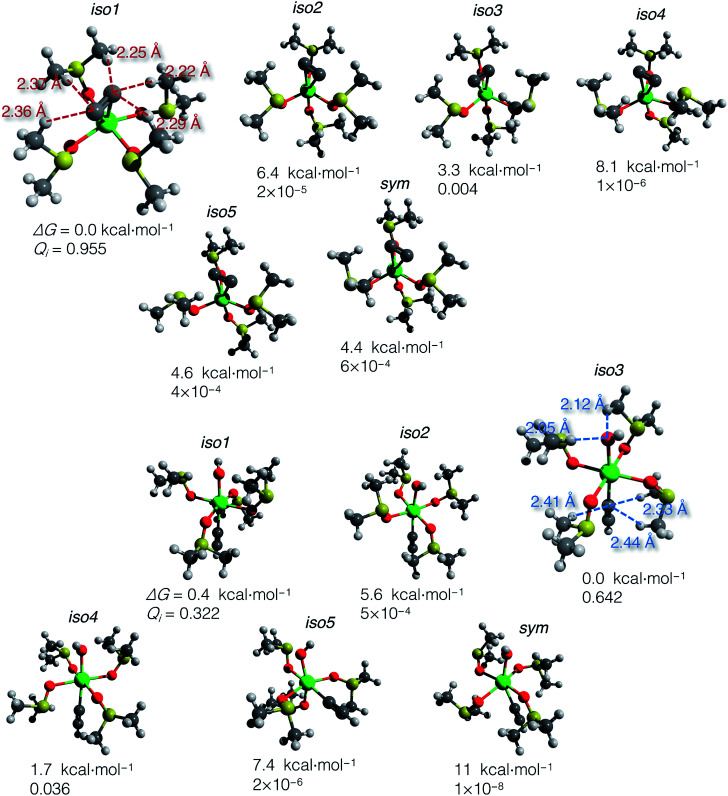
Optimized structures of conformers: [(DMSO)_4_Ca(C≡C)] (top) and [(DMSO)_4_Ca(C≡CH)(OH)] (bottom). Relative Gibbs energies and Boltzmann weights at 300 K are given below the structures. The most abundant conformers **iso1** and **iso3** are depicted with marked close noncovalent C(sp)–H and O–H contacts. Note that the sum of the van der Waals radii for the C(sp)–H and O–H contacts is 2.88 and 2.62 Å, according to Bondi.^70^

Using the gas-phase optimized geometries, we calculated Δ*G*_solv_ for every conformer structure within the SMD approach (Solvation Model based on Density). We listed relative Δ*G* of the conformers and the corresponding *Q*_*i*_ values in the ESI .xlsx table.† The most populated states (those with the highest *Q*_*i*_) in DMSO and vacuum mostly do not coincide; in all cases except **iso1** of [(DMSO)_4_Ca(C≡CH) (OH)] (shown in Fig. 4), the highest *Q*_*i*_-conformers in DMSO are minor in a vacuum. Such a discrepancy can be expected because polar DMSO stabilizes polar conformations of the solute.

The computed Δ*G* of the conformers in DMSO, the corresponding Boltzmann weights, and the optimized structures are given in Fig. 4. The relative free energies of conformers vary within 8.1 kcal mol^−1^ for [(DMSO)_4_Ca(C≡C)], and 11 kcal mol^−1^ for [(DMSO)_4_Ca(C≡CH)(OH)].

The DMSO molecules of the solvation shells can form hydrogen bonds with C_2_^2−^, HC≡C^−^, and OH^−^ ligands, thereby giving this considerable spread in relative Δ*G* in solution with the selected model systems and at the chosen level of theory. Close C–H and O–H contacts, as well as the reference sum of the van der Waals radii, are given in Fig. 4.

In contrast to the case of [(DMSO)_4_Ca(C≡C)] that is predominantly represented by **iso1**, the model conformer space of [(DMSO)_4_Ca(C≡CH)(OH)] has two significant structures **iso1** and **iso3**, and the somewhat minor **iso4**. All this emphasizes the importance of conformational sampling for cluster-continuum modeling of species in solutions.

Boltzmann averaging over the conformers negligibly shifts the Gibbs energy of the ensemble of [(DMSO)_4_Ca(C≡C)] by 0.02 kcal mol^−1^, relative to the lowest energy conformer **iso1**. Similarly, the ensemble-averaged Gibbs energy of [(DMSO)_4_Ca(C≡CH)(OH)] is 0.19 kcal mol^−1^ higher than that of **iso3**. Even though the averaging correction at 300 K is minor, we still suggest using the presented two-step conformational sampling (BOMD plus static DFT). Therefore, in the absence of the sampling, if one considers only a minor conformer with low *Q*_*i*_, Δ*G* of elementary reaction steps can be inaccurate by several kcal mol^−1^.

Now we can estimate Δ*G* of the following reactions using the averaged free energies of the solvated species:1
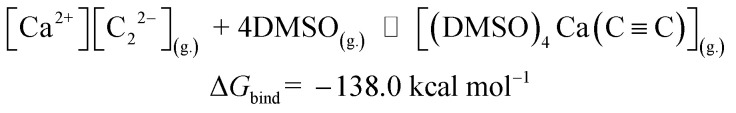
2
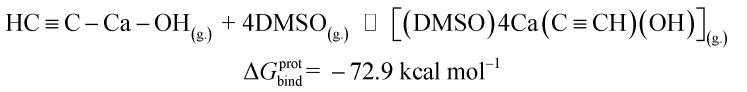


The reactions in eqn (1) and (2) are among the model steps in Fig. 2. We attribute the extremely exergonic effect of reactions (1) and (2) to the formation of strong Ca–O bonds, and—equally importantly—to the formation of many hydrogen bonds in the solvation shell. Even anionic centers of HC≡C^−^ and C≡C^2−^ ligands are hydrogen bond acceptors, as can be seen from the abundance of close contacts in the structures in Fig. 4.

The last step in Fig. 2 is to compute solvation energies of [(DMSO)_4_Ca(C≡C)] and [(DMSO)_4_Ca(C≡CH)(OH)] (see eqn (3) and (4) below). The process of the immersion of electro-neutral species [(DMSO)_4_Ca(C≡C)] and [(DMSO)_4_Ca(C≡CH)(OH)] into DMSO is moderately exergonic, in contrast to the gas-phase formation of the coordination shell, as in eqn (1) and (2).3
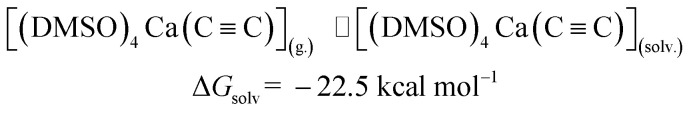
4
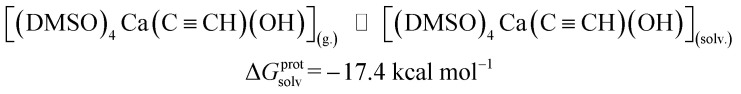


In this work, we selected M06-2X/6-31+G** as the underlying method for SMD computations of Δ*G*_solv_ since 6-31+G**^71^ was included in the original SMD parameterization,^72^ and since this CSM is often used in conjunction^73–75^ with the M06-2X functional.^76^ In Section S4,† we demonstrate that predictions of Δ*G*_solv_ with SMD at the M06-2X/6-31+G** level deviate by only 0.7 kcal mol^−1^ from those obtained at the M05-2X/6-31+G** level that was used in the original parameterization of SMD.^72^

A closely related two-step model process is the hydration of Ca^2+^ (Table 1). The details of the performed modeling of Ca^2+^ solvation in water are described in the ESI table.† As in the previous case with DMSO, most of the solvation exergonicity stems from the formation of the coordination sphere. The experimental value for the hydration of Ca^2+^ in water varies from −359.7 (ref. 77) to −386.2 (ref. 78) kcal mol^−1^ (the divergence is equal to 26.5 kcal mol^−1^), so the comparison with the experiment is possible, but cannot be performed reliably. Depending on the experimental reference, our computational estimation of Δ*G*_solv_ deviates from −1.3 to −27.8 kcal mol^−1^. The continuum models, used directly, *i.e.*, without the explicit inclusion of a solvation shell, yield minimal deviations of +66.0, +64.7, and +75.9 kcal mol^−1^ for the COnductor-like Screening MOdel (COSMO), conductor-like polarizable continuum model (C-PCM), and SMD, respectively. Cluster-continuum computations, with our two-step calculation of Δ*G*_solv_ in H_2_O being one of this class, are a well-established approach to the modeling of ionic species in solution.^79–82^

**Table 1:** Hydration of Ca^2+^^a^

Transformation	Δ*G*_rxn_, kcal mol^−1^
Ca_(g.)_^2+^ + 7H_2_O_(g.)_ ⇌ [Ca(H_2_O)_7_]_(g.)_^2+^	−205.6
[Ca(H_2_O)_7_]_(g.)_^2+^ ⇌ [Ca(H_2_O)_7_]_(aq.)_2+	−181.9
Ca_(g.)_^2+^ + 7H_2_O_(g.)_ ⇌ [Ca(H_2_O)_7_]_(aq.)_^2+^	−387.5
Experimental reference	−386.2 (ref. 78) to −359.7 (ref. 77)
Classical (non-quantum) electrostatic models	−377.3,^83^ −403.2 (ref. 78)

a The binding of H_2_O to Ca^2+^ was modeled at the RIJK-PBE0-D3(BJ)/def2-TZVP-gCP level; the hydration was modeled using SMD (M06-2X/6-31+G**).

### Understanding CaC_2_ solubilization: direct solvation *vs.* hydrolytic solubilization

Table 2 summarizes the cumulative thermodynamic effect of the CaC_2_ dissolution in pure DMSO (+25.1 kcal mol^−1^) and its favorable hydrolysis in the DMSO/water solvent system (−9.1 kcal mol^−1^). The former is markedly endergonic, in accordance with the experimental observations of CaC_2_ inactivity in pure DMSO.^19,21,23,35,36^ Therefore, CaC_2_ dissolution in DMSO is thermodynamically forbidden. The solubilization can be achieved *via* the steady protonation of CaC_2_ at the solid–liquid interface, and the concomitantly formed HC≡C^−^ can participate in subsequent transformations.

**Table 2:** Summary: the unfavorable CaC_2_ solvation *vs.* protolysis-assisted solubilization of CaC_2_^a^

Transformation	Δ*G*_rxn_, kcal mol^−1^
**Direct solvation**	
CaC_2(s.)_ ⇌ [Ca^2+^][C_2_^2−^]_(g.)_	Δ*G*_sub_ = 185.6
[Ca^2+^][C_2_^2−^]_(g.)_ + DMSO_(g.)_ ⇌ [(DMSO)_4_Ca(C≡C)]_(g.)_	Δ*G*_bind_ = −138.0
[(DMSO)_4_Ca(C≡C)]_(g.)_ ⇌ [(DMSO)_4_Ca(C≡C)]_(solv.)_	Δ*G*_solv_ = −22.5
CaC_2(s.)_ ⇌ CaC_2(solv.)_ (same as [(DMSO)_4_Ca(C≡C)]_(solv.)_)	Δ*G*_sub_ + Δ*G*_bind_ + Δ*G*_solv_ = 25.1

**Protolysis-assisted solubilization**	
[Ca^2+^][C_2_^2−^]_(g.)_ ⇌ HC≡C–Ca–OH_(g.)_	Δ*G*_prot_ = −104.5
HC≡C–Ca–OH_(g.)_ + 4 DMSO_(g.)_ ⇌ [(DMSO)_4_Ca(C≡CH)(OH)]_(g.)_	Δ*G*^prot^_bind_ = −72.9
[(DMSO)_4_Ca(C≡CH) (OH)]_(g.)_ ⇌ [(DMSO)_4_Ca(C≡CH)(OH)]_(solv.)_	Δ*G*^prot^_solv_ = −17.4
CaC_2(s.)_ ⇌ HC≡C–Ca–OH_(solv.)_ (same as [(DMSO)_4_Ca(C≡CH) (OH)]_(solv.)_)	Δ*G*_sub_ + Δ*G*_prot_ + Δ*G*_bind_^H+^ + Δ*G*_solv_^H+^ = −9.1

a Gas-phase thermochemistry was modeled at the PBE0-D3(BJ)/pob-TZVP-gCP level; bulk solvent effects were modeled using SMD (M06-2X/6-31+G**).

H_2_O can easily protonate C_2_^2−^, as it is a much stronger acid. At the same time, H_2_O is less acidic than HC≡CH in DMSO/water solutions, as seen from Table 3. We used a coarse quantum chemical approach to calculate free energies of H_2_O, DMSO, HC≡CH, and PhC≡CH deprotonation in DMSO (see also the ESI .xlsx table†). The reference p*K*_a_ values show that H_2_O is ∼10^2^ times less acidic than HC≡CH, and ∼10^10^ times less acidic according to our calculations. The deprotonation of HC≡C^−^ yielding C_2_^2−^ should be as unfavorable as DMSO autoprotolysis.

**Table 3:** Acidity in DMSO

Transformation	Δ*G*_rxn_, kcal mol^−1^	Calculated p*K*_a_	Reference p*K*_a_	Deviation^a^
HC≡CH_(solv.)_ ⇌ HC≡C_(solv.)_^−^ + H_(solv.)_^+^^b^	34.2	25.1	29.7^84^	−4.6
HC≡C_(solv.)_^−^ ⇌ ^−^C≡C_(solv.)_^−^ + H_(solv.)_^+^	50.6	37.1	—	
PhC≡CH_(solv.)_ ⇌ PhC≡C_(solv.)_^−^ + H_(solv.)_^+^	34.6	25.4	28.7^85^	−3.6
H_2_O_(solv.)_ ⇌ HO_(solv.)_^−^ + H_(solv.)_^+^	48.8	35.8	31.4^86^	4.4
CH_3_S(O)CH_3(solv.)_ ⇌ CH_3_S(O)CH_2(solv.)_^−^ + H_(solv.)_^+^	50.5	37.0	35.1^86^	1.9

a Between calculated and reference values.

b The Gibbs free energy of a proton in DMSO is taken as the sum of G in the gas phase at 298.15 K and 1 atm (ref. 87) and Δ*G*_solv_ of H^+^ in DMSO.^88^

Acidities (p*K*_a_) of DMSO and HC≡C^−^ (second stage) are nearly equal, according to the PBE0/ma-def2-TZVP + SMD calculation. Therefore, we may suppose DMSO as a possible protolytic agent for C_2_^2−^ in solution. Indeed, C_2_^2−^ anions can undergo rapid protonation by DMSO (see the ESI .xlsx table†), meaning that the formation of free acetylide dianions in such a solution system is hardly possible. The solvent is not aprotic enough, even if we find a way to effectively solvate C_2_^2−^ with anion-sequestering host molecules, *e.g.*, cavitands.

Modeled at the PBE0/ma-def2-TZVP level; bulk solvent effects were accounted for by applying SMD (M06-2X/6-31+G**) (see Section S1.3† for details).

**Scheme 1:**
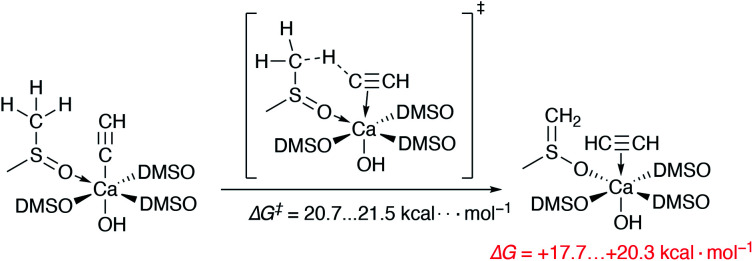
The unfavorable process of the HC≡C^−^ protonation by a DMSO molecule from the first solvation shell. All DMSO molecules are coordinated *via* O atoms.

We also estimated the favorability of HC≡C^−^ protonation by the DMSO molecules of the Ca^2+^ solvation shell (as in Scheme 1). The free energies of activation of the two evaluated pathways are 20.7 and 21.5 kcal mol^−1^. Moreover, the process is endergonic by 17.7–20.3 kcal mol^−1^. Thus, the protonation of the acetylide in [(DMSO)_4_Ca(C≡CH)(OH)] is somewhat kinetically unfavorable, also being clearly unfavorable thermodynamically.

Other protic molecules such as inorganic acids HX, HClO_4_, and CF_3_SO_3_H (X = Cl, Br, I) are an inappropriate choice for the protolytic activation of CaC_2_. These acids are reported to be strong in DMSO.^89^ That is why their DMSO solutions can protonate not only C≡C^2−^ but also HC≡C^−^, thereby decomposing the reactive acetylide intermediate. Moderate acidity is crucial in our case.

## Summary, conclusions, and outlook

A protolytic agent plays a crucial role in the activation of a carbide in solution reactions. Water is a unique agent since it is less acidic than HC≡CH in DMSO. The formation of anionic monoacetylide intermediates is the way to activate CaC_2_ in liquid-phase organic transformations. The dynamics of CaC_2(s.)_ protolysis, proceeding at the solid–liquid interface, may thus be of paramount importance for further understanding of CaC_2_ activation. Research on the dynamics of this interface process is currently underway in our group.

We tested a new modeling strategy for solvated ionic pairs formed in the process of dissolution or protolysis of ionic crystals. It allowed us to obtain ensemble-averaged Δ*G* of reactions in solutions with species for which no CSM parameterization is available. We plan to further use and test this computational methodology, as well as encourage its use in other groups.

The methodology is modular, as it consists of three distinct steps depicted in Fig. 1. Therefore, evaluating alternative tight-binding parameterizations (*e.g.*, eXtended Tight-Binding methods and GFN*n*-xTB)^90,91^ and CSMs (such as COSMO-RS)^92^ is advised to determine an optimal level of theory. Accounting for anharmonic effects in calculations of free energies can be another option for incremental improvement of the methodology. Such effects can be incorporated in the solid state step (Fig. 1, left),^93,94^ as well as in the MD^95,96^ and molecular DFT steps^97,98^ (Fig. 1, middle and right, respectively), although in the latter two cases this may be technically non-trivial. It was shown in recent studies of solid state and surface systems that anharmonic effects may be crucial.^99,100^ However, we should also mention a critique of existing approximations for computation of anharmonic free energies.^101^

Strongly coordinating solvents such as DMSO form a well-defined solvation shell that should be sampled with BOMD. A very economical choice is to use a tight-binding Hamiltonian such as DFTB3 with empirical corrections for non-covalent interactions. In our case, running even relatively short equilibration trajectories of 10 ps yielded ensemble-sampled structures of solvation shells. Free energy computations with MD methods require rather elaborate techniques.^102,103^ That is why Boltzmann averaging over an MD-obtained set of solvation shell conformers can be a convenient option. The proposed combination of semi-empirical BOMD and static DFT computations of Δ*G* values is cost-efficient since the most demanding step—the sampling of conformer space with MD—is feasible even on a personal workstation. We performed most of the MD simulations on an entry-level graphics processing unit (GPU) and a gaming central processor (CPU, see the ESI† for details).

As a fundamental result, we propose a strategy for CaC_2_ activation in organic media that can boost further development of green and sustainable synthetic methodologies based on the use of calcium carbide. DMSO, as well as dimethylformamide which is widely used in reactions with CaC_2_, is not a particularly “green” solvent. Less toxic polar aprotic solvents that allow water p*K*_a_ higher than acetylene p*K*_a_ would be a better choice for future organic synthesis; no less important is the propensity to effectively solvate Ca^2+^ by forming strong Ca-solvent bonds, such as, *e.g.*, Ca–O. There are few such solvents. Here we assessed H_2_O as a suitable green protolytic agent for a solid acetylide. However, we hypothesize that any molecule less protic then HC≡CH in a given solvent can play its role, thereby allowing new synthetic transformations. Computational methods, as described in this work, can help in the evaluation of known green solvents for sustainable organic synthesis with CaC_2_ or in the search for new ones, as well as in the discovery of new protolytic agents for the activation of CaC_2_.

## Computational details

Solvation free energies of species in Fig. 2 were estimated using ORCA 4.1.2.^104^ The solvation model based on density (SMD) was selected for this purpose.^72^ All implicit solvent computations were performed on gas-phase geometries, as in the original studies.^72,105,106^ Δ*G*_solv_ is the difference between the single point (total) energies of gas-phase geometries with SMD applied, and without. We chose the diffuse basis set (BS) 6-31+G**,^71,107,108^ since we modeled anionic species in p*K*_a_ estimations, and, at the same time, this basis set was included in the SMD parameterization. For Ca, we accepted the default ORCA^104^ choice and used diffuse exponents from 6-311+G**, which was adopted from the EMSL basis set exchange.^109–111^ In the original work, SMD was parameterized for use at the M05-2X/6-31+G** level of theory;^112^ the corresponding functional is, however, unavailable in ORCA. Therefore, we selected its successor, M06-2X,^76^ that is successfully employed in computations with SMD^73–75^ (see also Sections S1.2 and S4†).

The CRYSTAL17 (ref. 113) program was used for evaluation of gas-phase energies and thermodynamic corrections for reactions in Fig. 2. The pob-TZVP basis set was used.^114^ The PBE0 functional was selected. Empirical corrections for dispersion interactions (D3, including the Becke–Johnson dumping function) and geometrical counterpoise corrections (gCP)^115,116^ were included (see Section S1.1† for details).

The self-consistent charge density-functional tight-binding method DFTB3 (ref. 117 and 118) was used for Born–Oppenheimer molecular dynamics of model DMSO solutions. The computations were performed in the DFTB+ program (ver. 19.1).^119^ The Third-Order Parametrization for Organic and Biological Systems (3OB) of SCC-DFTB was used.^120–122^ All parameters selected in SCC-DFTB3 computations are given in Section S1.6,† together with a description of how model systems with explicit DMSO solvent were constructed.

We modeled C≡C^2−^ and ^–^C≡CH protonation by DMSO using the B97-3c method^123^ for gas-phase calculations and SMD for the evaluation of solvation free energies (as described above). These computations were performed with ORCA 4.1.2 (see Section S1.5† for details).

Travis (update Jan 01, 2019)^124^ was used to plot radial distribution functions.

## Conflicts of interest

There are no conflicts to declare.

## Supplementary Material

SC-011-D0SC04752J-s001

SC-011-D0SC04752J-s002

SC-011-D0SC04752J-s003

SC-011-D0SC04752J-s004

SC-011-D0SC04752J-s005

SC-011-D0SC04752J-s006

SC-011-D0SC04752J-s007

SC-011-D0SC04752J-s008

SC-011-D0SC04752J-s009

SC-011-D0SC04752J-s010

SC-011-D0SC04752J-s011

SC-011-D0SC04752J-s012

SC-011-D0SC04752J-s013

SC-011-D0SC04752J-s014

SC-011-D0SC04752J-s015

SC-011-D0SC04752J-s016

SC-011-D0SC04752J-s017

SC-011-D0SC04752J-s018

SC-011-D0SC04752J-s019

SC-011-D0SC04752J-s020

SC-011-D0SC04752J-s021

SC-011-D0SC04752J-s022

SC-011-D0SC04752J-s023

SC-011-D0SC04752J-s024

SC-011-D0SC04752J-s025

SC-011-D0SC04752J-s026

SC-011-D0SC04752J-s027

SC-011-D0SC04752J-s028

SC-011-D0SC04752J-s029

SC-011-D0SC04752J-s030

SC-011-D0SC04752J-s031

SC-011-D0SC04752J-s032

SC-011-D0SC04752J-s033

SC-011-D0SC04752J-s034

SC-011-D0SC04752J-s035

SC-011-D0SC04752J-s036

SC-011-D0SC04752J-s037

SC-011-D0SC04752J-s038

SC-011-D0SC04752J-s039

SC-011-D0SC04752J-s040

SC-011-D0SC04752J-s041

SC-011-D0SC04752J-s042

SC-011-D0SC04752J-s043

SC-011-D0SC04752J-s044

SC-011-D0SC04752J-s045

SC-011-D0SC04752J-s046

SC-011-D0SC04752J-s047

SC-011-D0SC04752J-s048

SC-011-D0SC04752J-s049

SC-011-D0SC04752J-s050

SC-011-D0SC04752J-s051

SC-011-D0SC04752J-s052

SC-011-D0SC04752J-s053

SC-011-D0SC04752J-s054

SC-011-D0SC04752J-s055

SC-011-D0SC04752J-s056

SC-011-D0SC04752J-s057

SC-011-D0SC04752J-s058

SC-011-D0SC04752J-s059

SC-011-D0SC04752J-s060

SC-011-D0SC04752J-s061

SC-011-D0SC04752J-s062

SC-011-D0SC04752J-s063

SC-011-D0SC04752J-s064

SC-011-D0SC04752J-s065

SC-011-D0SC04752J-s066

SC-011-D0SC04752J-s067

SC-011-D0SC04752J-s068

SC-011-D0SC04752J-s069

SC-011-D0SC04752J-s070

SC-011-D0SC04752J-s071

SC-011-D0SC04752J-s072

SC-011-D0SC04752J-s073
