# Supplementary material for: Understanding the solubilization of Ca acetylide with a new computational model for ionic pairs
Source: Chem Sci. 2020 Oct 8;11(48):13102–12. doi: 10.1039/d0sc04752j (PMC8163204; doi:10.1039/d0sc04752j)
Supplement: SC-011-D0SC04752J-s073 [file SC-011-D0SC04752J-s073.pdf]

Electronic Supplementary Material (ESI) for Chemical Science.
This journal is © The Royal Society of Chemistry 2020

Supporting Information for the Article

Understanding Solubilization of Ca Acetylide with a New Computational Model for Ionic Pairs

Mikhail V. Polynski†‡, Mariia D. Sapova,† Valentine P. Ananikov*†‡*

† Saint Petersburg State University, Universitetsky Prospect 26, Saint Petersburg 198504, Russia.

‡ Zelinsky Institute of Organic Chemistry, Russian Academy of Sciences, Leninsky Prospekt 47, Moscow 119991, Russia.

AUTHOR INFORMATION

* polynskimikhail@gmail.com (M.V.P.); val@ioc.ac.ru (V.P.A.).

Table of Contents

1. Computational Details 3

1.1. Solid State and Gas-phase CaC_2 3

1.2. ΔG_{solv} Computations with CSMs 4

1.3. Coarse pK_a Computations 5

1.4. Ca^{2+} Hydration 5

1.5. Mechanisms of $C{\equiv}C^{2-}$ and $^{-}C{\equiv}CH$ Protonation by DMSO 6

1.6. DFTB Modeling of DMSO Solutions 7

2. Modeling CaC_2 Polymorphs and Clusters 9

2.1. Relative Stability of CaC_2 Polymorphs 9

2.2. Unfavorable Sublimation of $CaC_{2(s.)}$ to $(CaC_2)_n$ Clusters 10

3. CaC_2 Ionic Pair in Implicit (Continuum) Solvent 12

4. Comparing SMD results at different levels of theory 13

5. Details of the MD runs 14

5.1. Equilibrating the $[Ca^{2+}][C_2^{2-}]$/DMSO system 15

5.2. Equilibrating the HC≡C-Ca-OH/DMSO system 17

5.3. Simulated annealing of the $[Ca^{2+}][C_2^{2-}]$/DMSO system 19

5.4. Sampling the HC≡C-Ca-OH/DMSO system 21

6. References 23

1. Computational Details

A notice should be given on calculations ΔG of reactions involving charged species in the gas phase. In this case, one needs to calculate molar Gibbs energies of naked cations or anions, which is only a hypothetical state of matter. It was suggested to use the formulae for the ideal gas as, for example, the Sackur-Tetrode equation.[1,2] Indeed, ΔG computations treating charged gas-phase species as the ideal gas are ubiquitous and offer well acceptable accuracy, compared to experiment.[3–6] Therefore, we used the IG approximation for gas-phase cations and anions in this work, while being inherently dissatisfied with such an approach.

Except for the computations described in Section S1.1, we run all calculations on a simple workstation PC with an Intel Core i7-9700K CPU overclocked to 5 GHz (gaming-grade CPU), 48 Gb of RAM, and an entry-level MSI GeForce GTX 1050 Ti GPU (latter was used for the computations in the DFTB+ program). Notably, two runs were performed most of the time simultaneously (on CPU and on GPU).

1.1. Solid State and Gas-phase CaC_2

All solid-state density functional theory (DFT) calculations were performed using the CRYSTAL17 software.[7] In CRYSTAL17, crystalline orbitals (CO) are approximated as a linear combination of Bloch sums of Gaussian type orbitals (GTOs). We used a triple-zeta valence basis set with polarization quality (pob-TZVP) optimized for solid-state calculations[8] in combination with the geometrical counterpoise correction (gCP).[9,10] We used the hybrid PBE0 functional[11] with Grimme's dispersion D3 correction, including three-body term and Becke-Johnson dumping function.[12,13]

Overlap thresholds for Coulomb and exchange integrals were tightened to 10^{-8}, 10^{-8}, 10^{-8}, 10^{-8}, and 10^{-16}. Integration over the Brillouin zone was performed on the Monkhorst–Pack grid with the shrinking factor 6 corresponding to the primitive tetragonal cell with inversely scaling to the cell constants. We performed full optimization of cell parameters and atomic displacements. For the self-consistent calculations (SCF) the tolerance on total energy change was set to 10^{-10} Hartree.

Gas-phase CaC_2 calculations were performed on the same level of theory as for the solid-state (PBE0-D3/pob-TZVP-gCP).

1.2. ΔG_{solv} Computations with CSMs

In all computations with CSMs applied, ΔG_{solv} is the difference between single point (total) energies of gas-phase geometries with a CSM applied, and without.

SMD and C-PCM computations were performed in ORCA 4.1.2. Tight SCF convergence criteria were set in all computations (the keyword TightSCF). A dense integration grid used for Kohn-Sham energy computations (GRID6, NOFINALGRID). Fock matrices were recalculated in each Kohn-Sham SCF iteration. See a detailed description of SMD calculation parameters in the main text. C-PCM computations were performed at the PBE0/def2-TZVP level of theory.[14]

Conventional COSMO[15] computations were performed in the ORCA 3.0.3 package. In this case, we employed the BP86 functional[16–18] and TZVP basis set[19] (the recommended choice for COSMO-RS calculations).[20] As long as the Resolution-of-the-Identity approximation (RI)[21–27] was used, the auxiliary basis set TZVP/J [25] was employed.

Additional computations were performed using the NWChem 7.0.0 package[28] within the restricted Kohn-Sham formalism to investigate how the choice of the underlying level of theory affects solvation free energies computed with SMD. The 6-31+G** basis set[29] from the NWChem

library was used. The M05-2X, M06-2X, and PBE0 were used to estimate ΔG_{solv} as the difference between single point (total) energies of gas-phase geometries with SMD applied, and without. The results are given in Section

1.3. Coarse pK_a Computations

We chose PBE0 functional and the diffuse ma-def2-TZVP basis set[30] for calculations of pK_a since deprotonated anions were modeled. The RI approximation was not used in this case. The convergence strategy "VerySlowConv" and tight convergence criteria (TightSCF) were used in the KS-SCF procedure. Fock matrices were recalculated in every KS-SCF iteration. A dense integration grid was used (GRID6, NOFINALGRID). Vibrational frequencies were computed numerically. The otherm program[31] provided by the Duarte research group was used for the free energy computations. Free Gibbs energies were calculated within the ideal gas-quantum rotor-harmonic oscillator model (IG-QRRHO)[32] at T = 298.15 K and P = 1 atm; ΔG_{solv} were computed with SMD (see the previous Subsection).

1.4. Ca^{2+} Hydration

Since we were unable to model systems with Ca^{2+} cations in the periodic CRYSTAL17 program, we had to use the ORCA program at the level of theory closely resembling that one used for the computations in CRYSTAL17. PBE0 functional was selected in conjunction with the def2-TZVP basis set.[14] The RI approximation was used for both Coulomb and exchange terms;[33,34] accordingly, the def2/JK basis set[35] was employed. The gCP empirical correction for the BSSE was used.[9] Also, we used D3-correction for dispersion interactions with the Becke-Johnson damping function, and three-body terms included.[12,13] The TightSCF, GRID6, and

NOFINALGRID options were set. Vibrational frequencies were computed numerically. Fock matrices were recalculated each 8-th KS-SCF iteration. See the "Ca2+ and HCC-Ca-OH" tab of the supporting .xslx table for the formulae used in the calculation of the Ca^{2+} Gibbs free energy.

1.5. Mechanisms of $C{\equiv}C^{2-}$ and $^{-}C{\equiv}CH$ Protonation by DMSO

This Subsection is relevant to the "Barriers" tab of the supporting .xslx table. For a rough estimation of free energy barriers of the protonation within the Born-Oppenheimer approximation, we selected the B97-3c method.[36] It is a combination of a re-parameterized B97[37] functional and empirical corrections.[12] In B97-3c, a specially optimized triple- ζ basis set is used. We performed the B97-3c computations within the RI approximation and used the default auxiliary basis set (def2-mTZVP/J).

To find the transition states of the proton transfer, first, relaxed potential surface scans were performed in which H^{+} migrated from the donor to acceptor atoms. Second, the highest energy structures from the scans were used for geometry optimization to transition states. All transition state structures exhibited a single imaginary mode corresponding to the proton vibration along the donor-acceptor line. The structures are included in the supporting PDF file in the XYZ format. Each .xyz file contains the value of the imaginary mode within it as a commentary line.

The bulk solvent effects we accounted for with SMD. The ΔG_{solv} values were computed for the stationary points and transition states, as described in Section S1.2.

1.6. DFTB Modeling of DMSO Solutions

The integration of the equations of motion was done with the velocity Verlet algorithm and timestep of 0.5 fs. The Berendsen thermostat and barostat[38] were used in the equilibration MD runs. The Nosé-Hoover chain thermostat (chain length equal to 3)[39] was used for sapling runs and in the simulated annealing (SA). Except in the SA run, the target temperature of the thermostat was set to 300 K, while the external target pressure was set to 10^5 Pa. No constraints on cell vectors and angles were applied during the NPT runs. Initial velocities in the equilibration runs were set according to the Boltzmann distribution at 300 K. In the sampling runs, velocities from the last iteration of the equilibration runs were used as initial.

During the equilibration runs, the coupling strength of the Berendsen thermostat, as well as that of the barostat, was controlled by setting the timescale parameter as equal to 100 fs. Simulated annealing was performed at the cell volume relaxed in the equilibration run (19.996 × 19.996 × 19.996 Å, $\alpha = \beta = \gamma = 90°$). The temperature profile for the SA is described in the main text; the coupling strength of the Nosé-Hoover chain thermostat was set to 10 THz. In the sampling run, the coupling strength of the Nosé-Hoover chain thermostat was 1 THz, and the coupling strength of the barostat was 1000 fs.

The SCC convergence tolerance was equal to 10^{-6} Hartree; modified Broyden's mixing scheme was used.[40] The parameter α in the Ewald electrostatic summation was determined automatically by the DFTB+. The tolerance for the Ewald summation was 10^{-8} Hartree. The MAGMA library (ver. 2.5.0)[41–45] was statically linked upon DFTB+ compilation to enable hybrid GPU-CPU computations. Orbital populations were assigned according to the Fermi function with the electronic temperature of 300 K; it should be noted that this low value of electronic temperature

led to integer populations in the insulating solution systems. Only Γ point was considered in electronic structure computations.

Hydrogen atom interactions were damped with the parameter ζ equal to 4.05.[46] Dispersion interactions were accounted for by the inclusion of the empirical corrections.[12,47]

The models of Ca(C≡C) and HO-Ca-C≡CH in DMSO were constructed in the following way. As a model structure of liquid DMSO, we took a box obtained via a classic MD simulation with the OPLS forcefield,[48] as provided at the "virtualchemistry.org" website.[49] We put the ionic pairs Ca(C≡C) and HO-Ca-C≡CH (pre-optimized at the PBE0-D3/pob-TZVP-gCP level) in the centers of periodic vacuum cells (2 × 2 × 2 nm each, one molecule per cell). The cells were filled with DMSO molecules using the `gmx solvate` utility of the GROMACS program package (ver. 2018.1)[50]; for this, van der Waals radii provided by Bondi[51] (for sp carbon only) and Batsanov (all other elements) were used.[52] The so-obtained cell with the first ionic pair, Ca(C≡C), contained 55 DMSO molecules. 54 DMSO molecules were included in the cell with the second ionic pair, HO-Ca-C≡CH.

In the partially hydrolyzed acetylide system, i.e., in HO-Ca-C≡CH in DMSO, we took equitemporal snapshots in the sampling run at the first MD step and then at exactly 2.5, 5.0, 7.5, and 10.0 ps. For the $[Ca^{2+}][C_2^{2-}]$ in DMSO, we took three snapshots in the simulated annealing run after the system cooled down to 300 K at 12, 14, and 16 ps. Also, in the $[Ca^{2+}][C_2^{2-}]$ system, we took two snapshots in the equilibration run after the thermodynamic parameters and the coordination number of Ca^{2+} equilibrated, at 7 and 9 ps.

2. Modeling CaC_2 Polymorphs and Clusters

2.1. Relative Stability of CaC_2 Polymorphs

According to previous research,[53–58] there are three co-existing phases of calcium carbide at room temperature: CaC_2-I (*I*4/*mmm*); CaC_2-II (*C*2/*c*); CaC_2-III (*C*2/*m*). The proportion of three polymorphic forms depends on the reaction conditions.[55]

Tetragonal phase CaC_2-I is prevalent at room temperature, but its thermodynamic stability remains uncertain as the previous theoretical works provide inconsistent results. CaC_2-I is stable according to earlier reports.[57,59,60] However, Häussermann, et al. calculated phonon dispersion of the tetragonal calcium carbide; it demonstrated dynamical instability at the X point of the Brillouin zone.[54] The authors considered two monoclinic phases (CaC_2-II, CaC_2-III) to be stable, with CaC_2-II being a low-temperature phase. In contrast, CaC_2-III phase is metastable according to the experimental work by Knapp and Ruschewitz.[55]

Doll, Jansen, et al. performed an extensive computational search for CaC_2 polymorphs.[58] Several new structures were predicted. CaC_2-I was found as particularly stable at standard pressure, as well as two of newly predicted polymorphs. Here we considered CaC_2-I, CaC_2-II, and CaC_2-III since these three phases are often observed in experimental studies.

We performed phonon calculations for the three CaC_2 modifications using appropriate supercells to consider multiple points of the Brillouin zone. Our computations indicate the dynamical stability of CaC_2-I (no imaginary frequencies at the X point was found) and CaC_2-II phases, while CaC_2-III has an imaginary frequency at Γ point. Relative electronic and Gibbs free energies of the phases are listed in Table S1. According to our calculations, CaC_2-I is the most stable polymorph. CIF files enclosing the primitive cells are provided in the SI.

We found no imaginary frequencies in the optimized supercell of CaC_2-I. Briefly, we used the Gaussian basis set pob-TZVP[8] instead of the plane-wave BS, the hybrid PBE0[11] functional instead of its pure GGA counterpart, and included corrections for BSSE[9,10] and dispersion interactions.[12,13] All computational parameters are given below.

Authors of Ref. [54] profoundly point out possible anharmonicity of phonon vibrations in CaC_2. As long as low-frequency modes in CaC_2 may indeed have anharmonic nature, considering CaC_2 phonon modes in anharmonic approximation seems worthwhile in future studies, as well as assessing relative stability of CaC_2 polymorphs with more sophisticated computational methods.

Gibbs free energy of the solid CaC_2 was averaged according to the Boltzmann distribution. The values of the Boltzmann weights Q_i demonstrate the equilibrium fractions of the CaC_2 polymorphs (see Table S1). CaC_2-I, as the most stable phase, is the most abundant in the equilibrium distribution.

Table S1. The relative electronic and Gibbs free energies of the CaC_2 polymorphs. Gibbs free energies and Boltzmann weights correspond to 300 K and 1 atm.

	ΔE_{el}, kJ/mol	ΔG (300 K), kJ/mol	Q_i (300 K)
CaC_2-I	0	0	0.89
CaC_2-II	3	7	0.11
CaC_2-III	11	14	0 (is a TS)

2.2. Unfavorable Sublimation of $CaC_{2(s.)}$ to $(CaC_2)_n$ Clusters

We performed calculations of a set of $(CaC_2)_n$ clusters with the number of formula units (n) ranging from 1 to 8 (see figures in the supporting .xslx table). All obtained clusters are dynamically

stable (no imaginary frequencies were observed). XYZ files with the cluster structures are provided in the supporting PDF file.

The Gibbs free energy of the cluster formation from crystal are presented in Table S2. We averaged the Gibbs free energy of the solid CaC_2 according to the Boltzmann distribution, as described above. Despite the energy of the cluster formation per formula unit decrease, the full energy increases significantly. Considering the fact that implicit solvation of molecules usually results in an exergonic effect of only 20-30 kcal/mol, we suggest crystal decomposition to the molecule CaC_2 (n=1) is by far the most favorable process.

Table S2. Free energies of degradation to clusters with a number of formula units z = 1-8.

z	Formula	Name	ΔG_{clust}, kJ/mol	$\Delta G_{clust}/n$, kJ/mol
1	CaC_2	CaC2_mol1_z1	777	777
2	$(CaC_2)_2$	CaC2_mol2_z2	994	497
2	$(CaC_2)_2$	CaC2_mol3_z2	988	494
2	$(CaC_2)_2$	CaC2_mol4_z2	945	472
3	$(CaC_2)_3$	CaC2_mol5_z3	1228	409
4	$(CaC_2)_4$	CaC2_mol6_z4	1604	401
4	$(CaC_2)_4$	CaC2_mol7_z4	1498	374
5	$(CaC_2)_5$	CaC2_mol8_z5	1774	355
6	$(CaC_2)_6$	CaC2_mol9_z6	1675	279
6	$(CaC_2)_6$	CaC2_mol10_z6	1615	269
6	$(CaC_2)_6$	CaC2_mol11_z6	2046	341
8	$(CaC_2)_8$	CaC2_mol12_z8	1981	248

3. CaC_2 Ionic Pair in Implicit (Continuum) Solvent

An overly simplified way is to model the solvation or hydrolysis of $CaC_{2(s.)}$ with a continuum solvation model (CSM). We calculated ΔG_{solv} of the acetylide species HC≡C-Ca-OH in DMSO with three CSMs, namely, COSMO,[15] C-PCM,[61] and SMD[62] (see Table S3 and the supporting .xslx table). It should be noted that neither of the used CSMs was initially parameterized for Ca systems. The more realistic approach, described in the main text, demonstrates that the exergonicity of the solvation of HC≡C-Ca-OH can be underestimated by CSMs by order of magnitude.

Table S3. ΔG_{solv} of $CaC_{2(g.)}$ in DMSO according to conventional continuum solvation models.

$[Ca^{2+}][C_2^{2-}]_{(g.)} \rightleftharpoons [Ca^{2+}][C_2^{2-}]_{(solv.)}$	
Method	ΔG_{solv}, kcal/mol
SMD (M06-2X/6-31+G**)	-19.1
C-PCM (PBE0/ma-def2-TZVP)	-25.2
COSMO (RI-BP86/TZVP)	-30.5

4. Comparing SMD results at different levels of theory

We checked, if ΔG_{solv} computed with SMD at the M06-2X/6-31+G** and PBE0/6-31+G** levels functionals are close to the results obtained at the M05-2X/6-31+G**, which was used in the original parameterization. ΔG_{solv} were calculated as the difference in total energies with and without SMD applied; gas-phase geometries optimized at the PBE0-D3(BJ)/pob-TZVP-gCP level were used for single point energy evaluations. Evidently, using SMD at the M06-2X/6-31+G** level leads to a small deviation of 0.7 kcal/mol on a small test set of related species. On the contrary, using PBE0/6-31+G** ΔG_{solv} estimations with SMD may lead to small but pronounced deviation of 1.7 kcal/mol, as shown in Table S4.

Table S4. Comparison of different DFT methods in SMD computations.

**Molecule**	**M05-2X**[a]	**M06-2X**[a]	**PBE0**[a]
$[(DMSO)_4Ca(C{\equiv}C)]$ *(iso1)*	-32.1	-31.2	-29.8
$[(DMSO)_4Ca(C{\equiv}CH)(OH)]$ *(iso3)*	-25.8	-24.8	-23.4
DMSO	-10.1	-9.7	-9.4
H_2O	-5.6	-5.5	-5.5
RMSD[b]	0	0.7	1.7

[a] The 6-31+G** basis set was used, see computational details in Section S1.2;

[b] Root-mean-square deviation, relative to the SMD at the original M05-2X/6-31+G** level;

All values are in kcal/mol. Corresponding full electronic energies are given in the supporting .xslx table.

5. Details of the MD runs

In this Section, plots of thermodynamic parameters (G, T, P, V) and radial distribution functions are given. Note, that for consistency with the definitions of the thermodynamic parameters in DFTB+, the following equation for G was used:

$$G = U + PV - TS_{electronic},$$

I.e., only electronic entropy was included.

5.1. Equilibrating the $[Ca^{2+}][C_2^{2-}]$/DMSO system

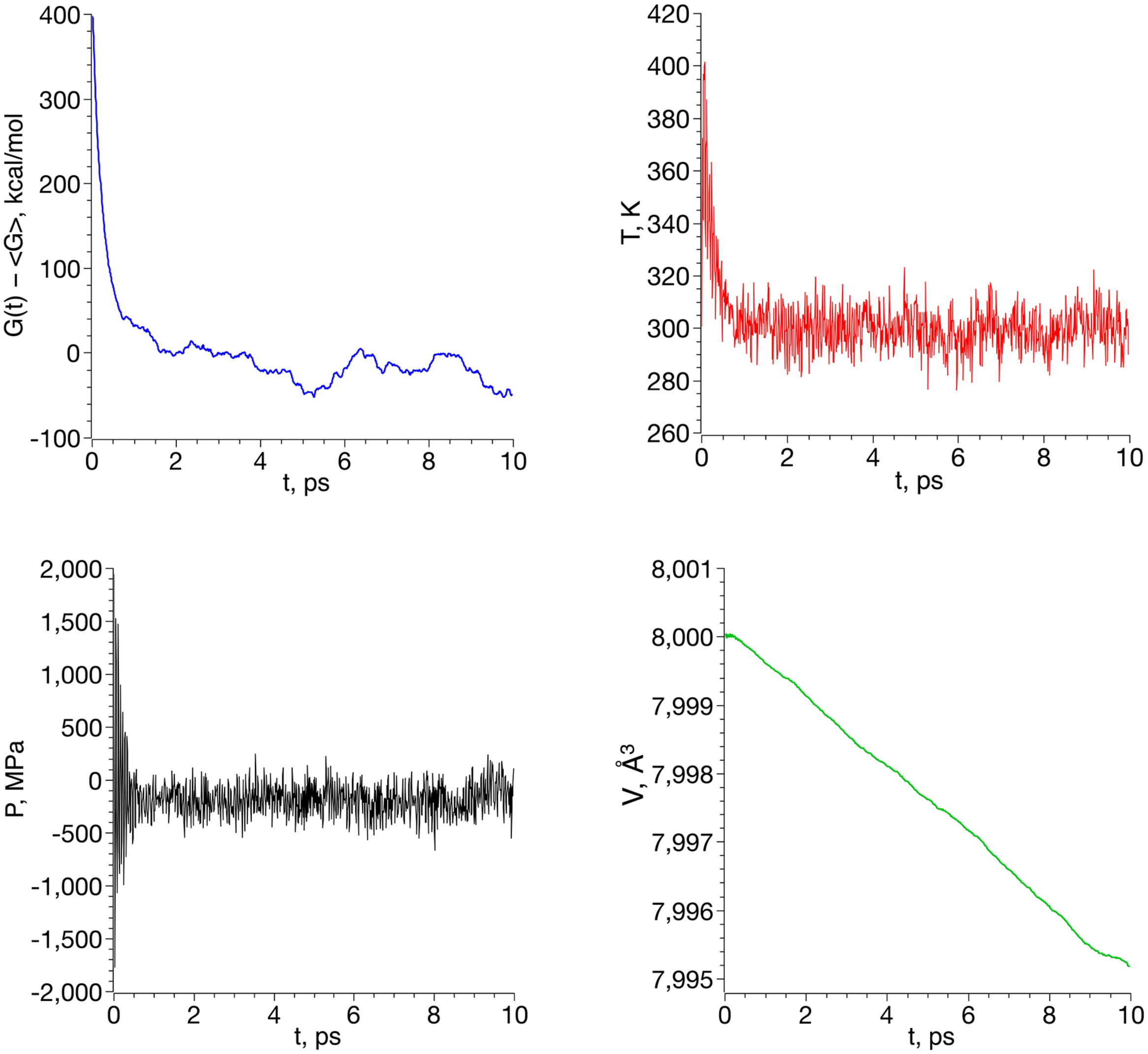

Figure S1. Thermodynamic parameters in the NPT simulation of $[Ca^{2+}][C_2^{2-}]$ in DMSO. Berendsen thermostat and barostat were used. $< G >$ is the mean value calculated on the entire trajectory.

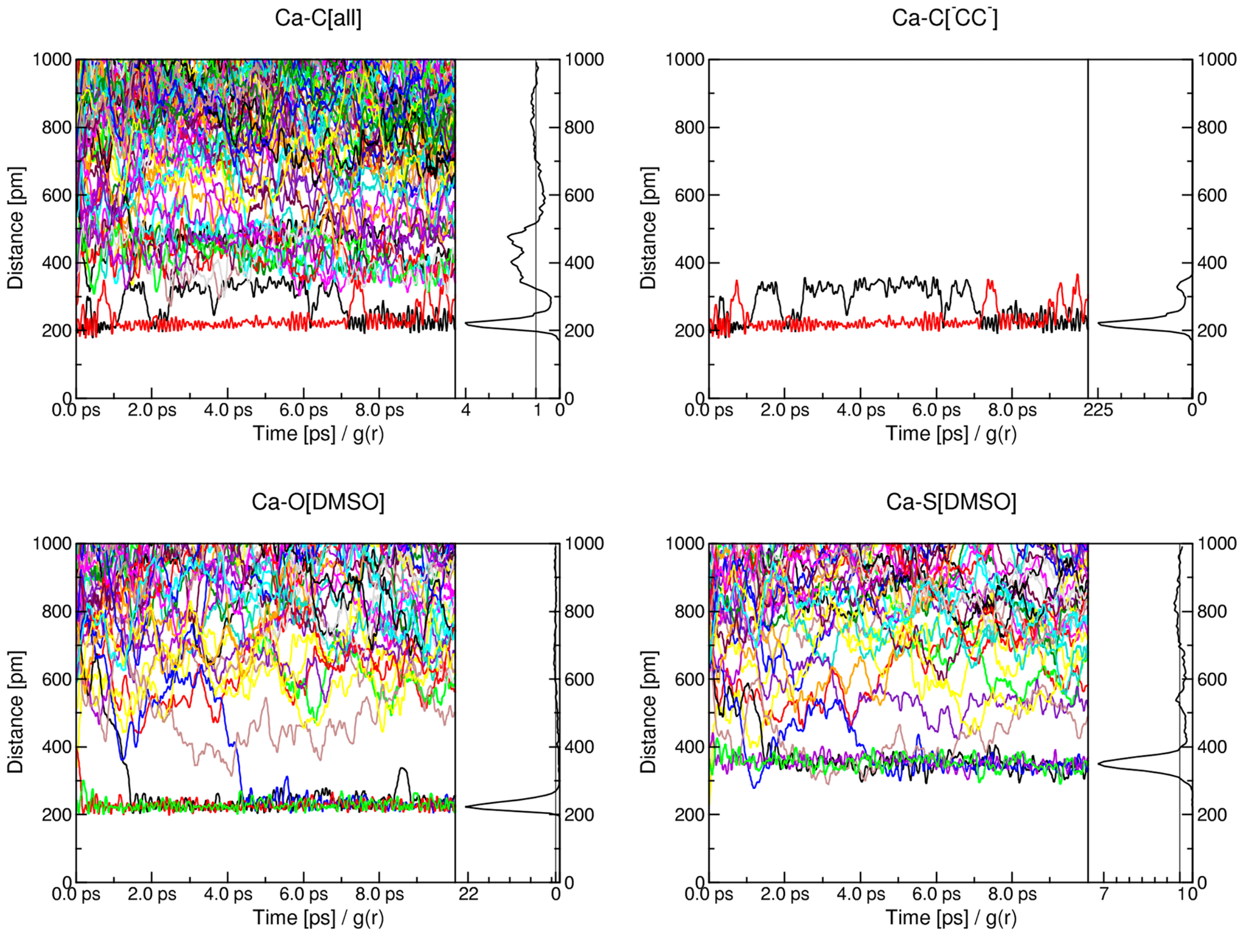

Figure S2. Radial distribution functions and evolution of selected interatomic distances in the equilibrating NPT simulation of $[Ca^{2+}][C_2^{2-}]$ in DMSO. Berendsen thermostat and barostat were used.

5.2. Equilibrating the HC≡C-Ca-OH/DMSO system

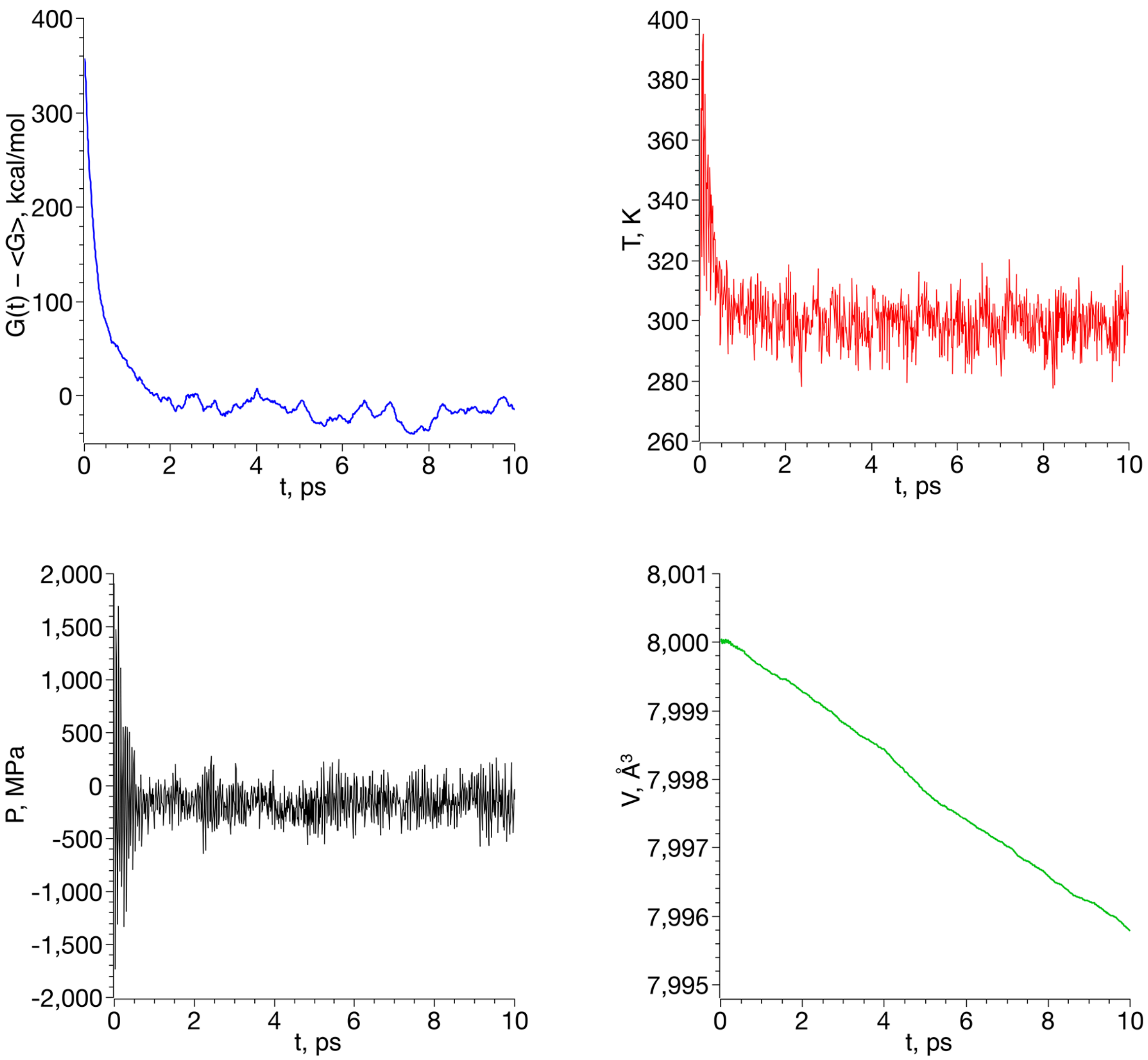

Figure S3. Thermodynamic parameters in the NPT simulation of HC≡C-Ca-OH in DMSO. Berendsen thermostat and barostat were used. $< G >$ is the mean value calculated on the entire trajectory.

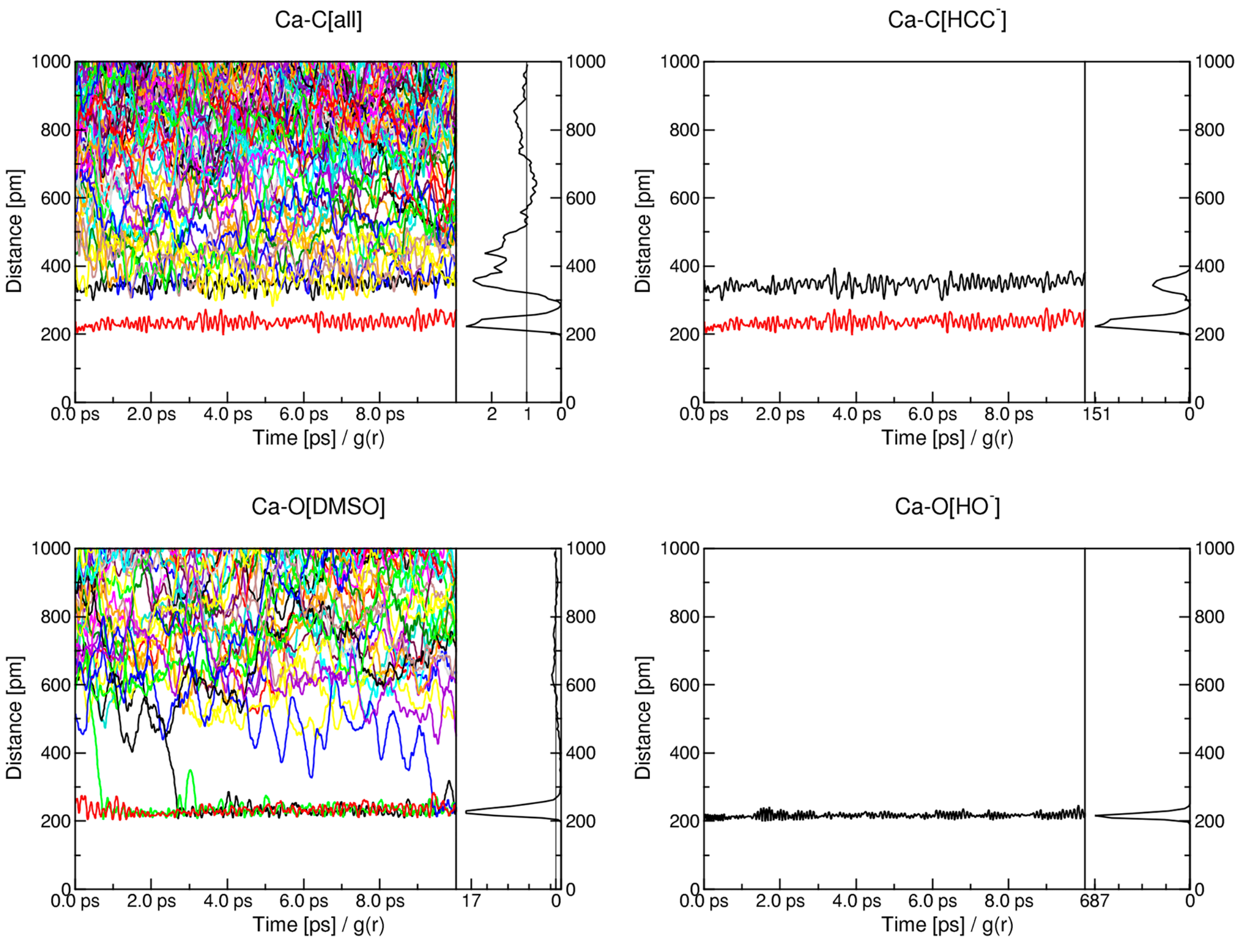

Figure S4. Radial distribution functions and evolution of selected interatomic distances in the equilibrating NPT simulation of HC≡C-Ca-OH in DMSO. Berendsen thermostat and barostat were used.

5.3. Simulated annealing of the $[Ca^{2+}][C_2^{2-}]$/DMSO system

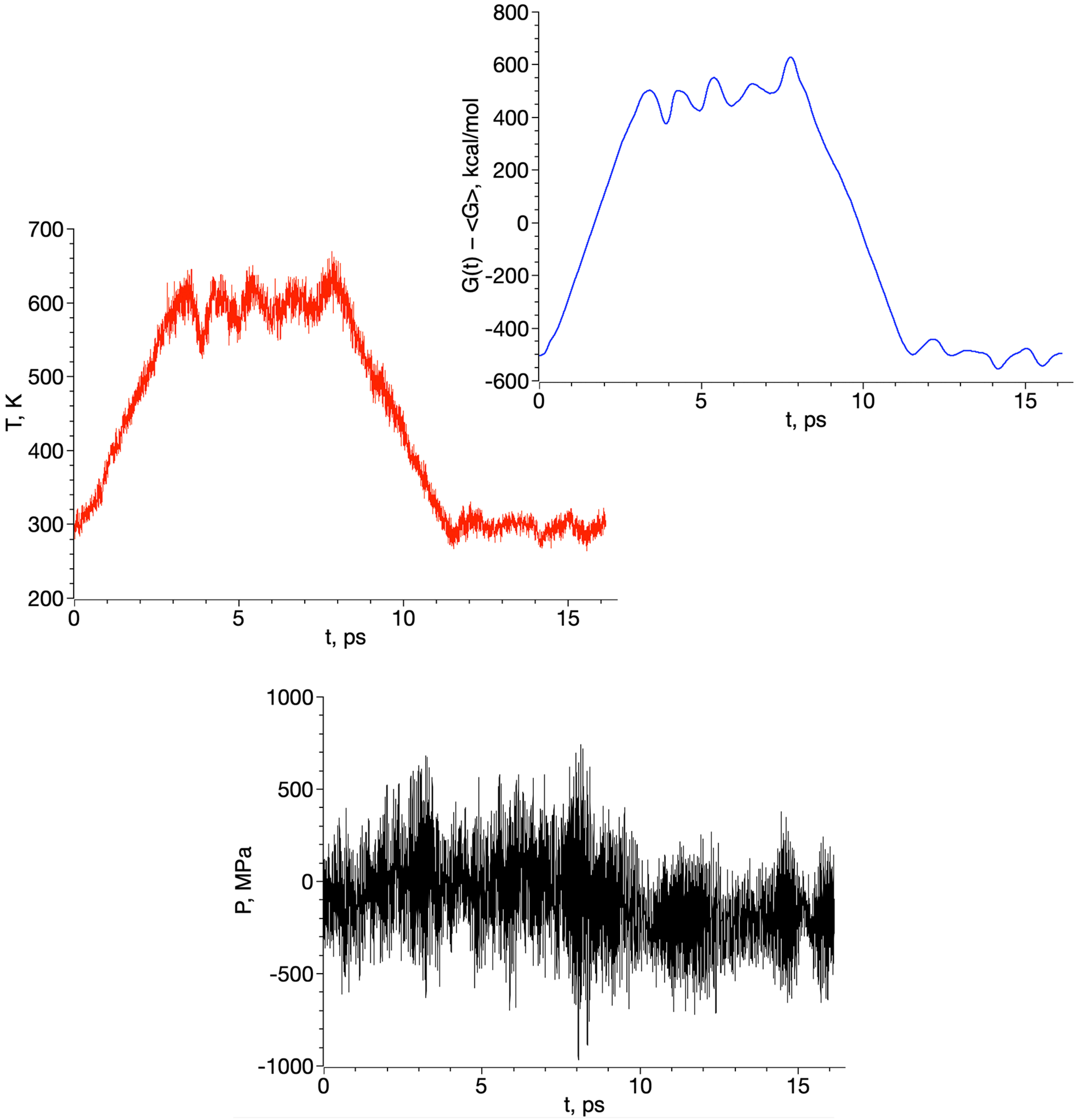

Figure S5. Thermodynamic parameters in the simulated annealing of $[Ca^{2+}][C_2^{2-}]$ in DMSO under NVT conditions. Berendsen thermostat and barostat were used. $< G >$ is the mean value calculated on the entire trajectory.

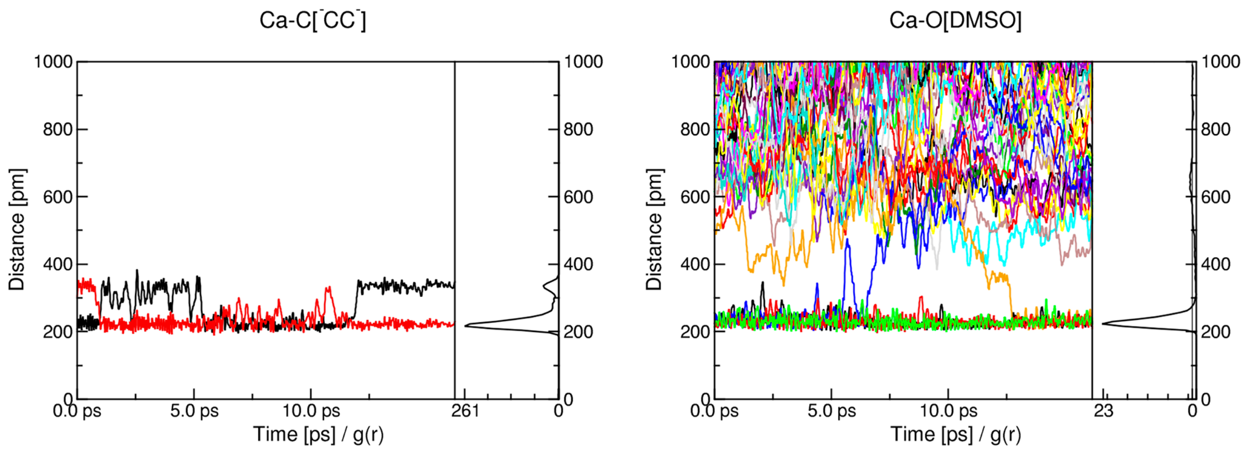

Figure S6. Radial distribution functions and evolution of selected interatomic distances in the simulated annealing of [Ca^{2+}][C_2^{2-}] in DMSO under NVT conditions. Berendsen thermostat and barostat were used.

5.4. Sampling the HC≡C-Ca-OH/DMSO system

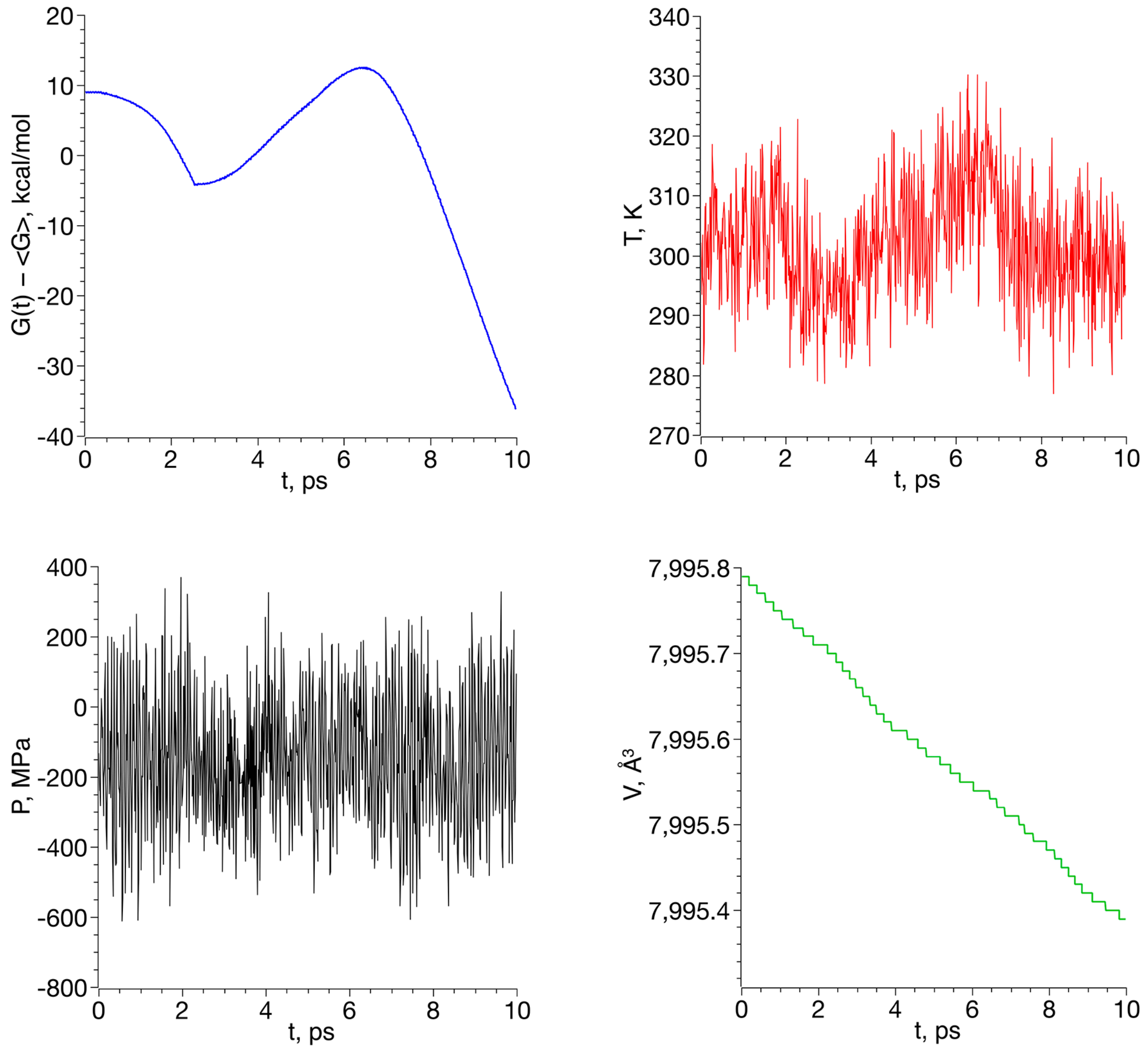

Figure S7. Thermodynamic parameters in the NPT simulation of HC≡C-Ca-OH in DMSO. Nosé-Hoover chain thermostat and Berendsen barostat were used. $< G >$ is the mean value calculated on the entire trajectory. Note the removable discontinuity in the $G(t) - < G >$ plot; this is the result of the forced simulation restart caused by a power outage. The simulation was restarted with the same atomic positions, velocities, and charges, but newly generated Nosé-Hoover chain variables.

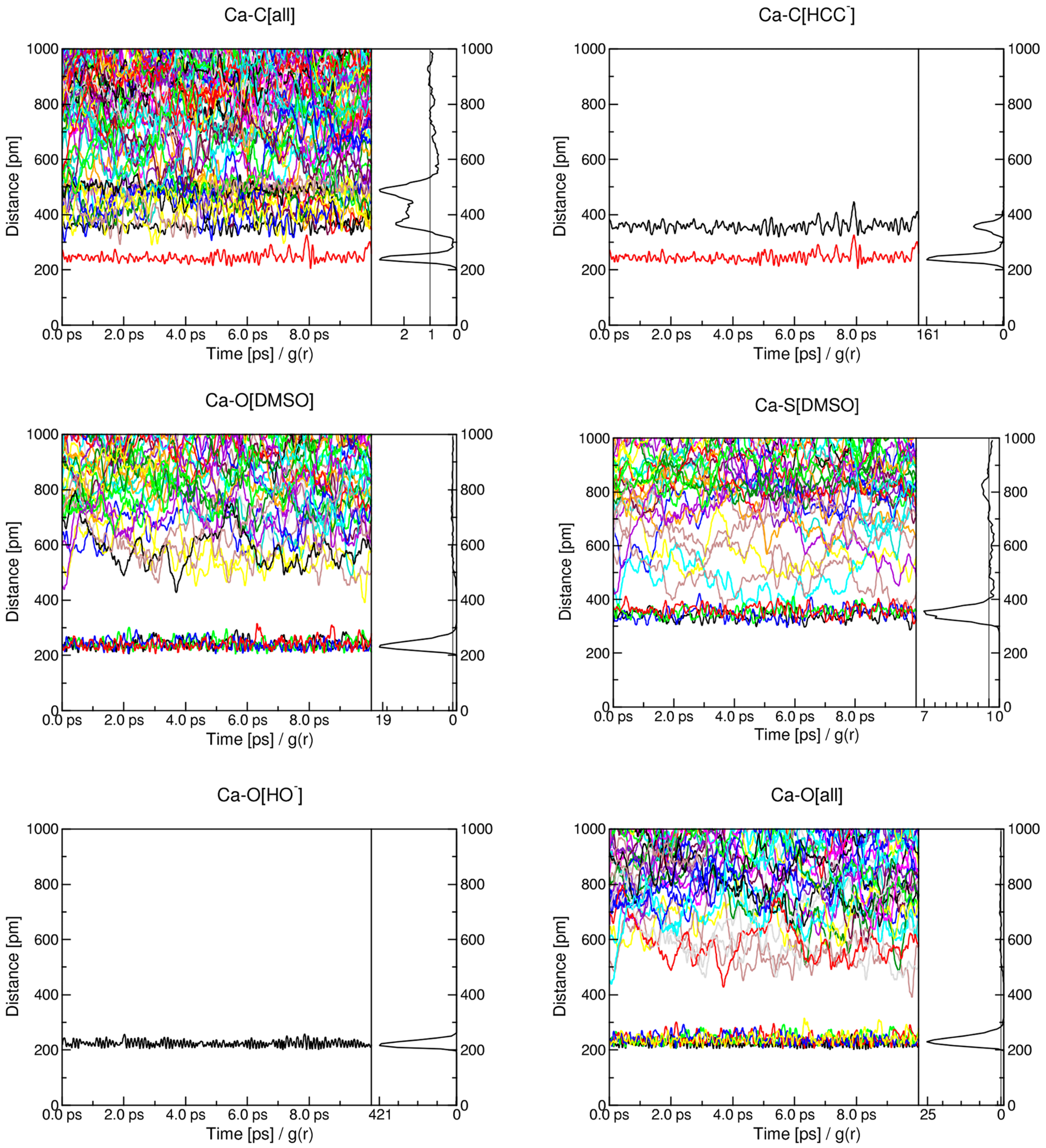

Figure S8. Radial distribution functions and evolution of selected interatomic distances in the sampling NPT simulation of HC≡C-Ca-OH in DMSO. Nosé-Hoover chain thermostat and Berendsen barostat were used.

6. References

1 C. P. Kelly, C. J. Cramer and D. G. Truhlar, *J. Phys. Chem. B*, 2007, **111**, 408–422.

2 A. Trummal, A. Rummel, E. Lippmaa, P. Burk and I. A. Koppel, *J. Phys. Chem. A*, 2009, **113**, 6206–6212.

3 J. R. Pliego and J. M. Riveros, *J. Phys. Chem. A*, 2001, **105**, 7241–7247.

4 Y. Takano and K. N. Houk, *J. Chem. Theory Comput.*, 2005, **1**, 70–77.

5 V. S. Bryantsev, M. S. Diallo and W. A. Goddard III, *J. Phys. Chem. B*, 2008, **112**, 9709–9719.

6 J. R. Pliego and J. M. Riveros, *Wiley Interdiscip. Rev. Comput. Mol. Sci.*, , DOI:10.1002/wcms.1440.

7 R. Dovesi, A. Erba, R. Orlando, C. M. Zicovich-Wilson, B. Civalleri, L. Maschio, M. Rérat, S. Casassa, J. Baima, S. Salustro and B. Kirtman, *Wiley Interdiscip. Rev. Comput. Mol. Sci.*, 2018, **8**, e1360.

8 M. F. Peintinger, D. V. Oliveira and T. Bredow, *J. Comput. Chem.*, 2013, **34**, 451–459.

9 H. Kruse and S. Grimme, *J. Chem. Phys.*, 2012, **136**, 154101.

10 J. G. Brandenburg, M. Alessio, B. Civalleri, M. F. Peintinger, T. Bredow and S. Grimme, *J. Phys. Chem. A*, 2013, **117**, 9282–9292.

11 C. Adamo and V. Barone, *J. Chem. Phys.*, 1999, **110**, 6158–6170.

12 S. Grimme, J. Antony, S. Ehrlich and H. Krieg, *J. Chem. Phys.*, 2010, **132**, 154104.

13 S. Grimme, S. Ehrlich and L. Goerigk, *J. Comput. Chem.*, 2011, **32**, 1456–1465.

14 F. Weigend and R. Ahlrichs, *Phys. Chem. Chem. Phys.*, 2005, **7**, 3297.

15 A. Klamt and G. Schüürmann, *J. Chem. Soc. Perkin Trans. 2*, 1993, 799–805.

16 S. H. Vosko, L. Wilk and M. Nusair, *Can. J. Phys.*, 1980, **58**, 1200–1211.

17 A. D. Becke, *Phys. Rev. A*, 1988, **38**, 3098–3100.

18 J. P. Perdew, *Phys. Rev. B*, 1986, **33**, 8822–8824.

19 A. Schäfer, H. Horn and R. Ahlrichs, *J. Chem. Phys.*, 1992, **97**, 2571–2577.

20 R. Franke and B. Hannebauer, *Phys. Chem. Chem. Phys.*, 2011, **13**, 21344–21350.

21 E. J. Baerends, D. E. Ellis and P. Ros, *Chem. Phys.*, 1973, **2**, 41–51.

22 B. I. Dunlap, J. W. D. Connolly and J. R. Sabin, *J. Chem. Phys.*, 1979, **71**, 3396.

23 C. Van Alsenoy, *J. Comput. Chem.*, 1988, **9**, 620–626.

24 R. A. Kendall and H. A. Früchtl, *Theor. Chim. Acta*, 1997, **97**, 158–163.

25 K. Eichkorn, F. Weigend, O. Treutler and R. Ahlrichs, *Theor. Chim. Acta*, 1997, **97**, 119–124.

26 K. Eichkorn, O. Treutler, H. Öhm, M. Häser and R. Ahlrichs, *Chem. Phys. Lett.*, 1995, **240**, 283–289.

27 J. L. Whitten, *J. Chem. Phys.*, 1973, **58**, 4496.

28 E. Aprà, E. J. Bylaska, W. A. de Jong, N. Govind, K. Kowalski, T. P. Straatsma, M. Valiev,

H. J. J. van Dam, Y. Alexeev, J. Anchell, V. Anisimov, F. W. Aquino, R. Atta-Fynn, J. Autschbach, N. P. Bauman, J. C. Becca, D. E. Bernholdt, K. Bhaskaran-Nair, S. Bogatko, P. Borowski, J. Boschen, J. Brabec, A. Bruner, E. Cauët, Y. Chen, G. N. Chuev, C. J. Cramer, J. Daily, M. J. O. Deegan, T. H. Dunning, M. Dupuis, K. G. Dyall, G. I. Fann, S. A. Fischer, A. Fonari, H. Früchtl, L. Gagliardi, J. Garza, N. Gawande, S. Ghosh, K. Glaesemann, A. W. Götz, J. Hammond, V. Helms, E. D. Hermes, K. Hirao, S. Hirata, M. Jacquelin, L. Jensen, B. G. Johnson, H. Jónsson, R. A. Kendall, M. Klemm, R. Kobayashi, V. Konkov, S. Krishnamoorthy, M. Krishnan, Z. Lin, R. D. Lins, R. J. Littlefield, A. J. Logsdail, K. Lopata, W. Ma, A. V. Marenich, J. Martin Del Campo, D. Mejia-Rodriguez, J. E. Moore, J. M. Mullin, T. Nakajima, D. R. Nascimento, J. A. Nichols, P. J. Nichols, J. Nieplocha, A. Otero-de-la-Roza, B. Palmer, A. Panyala, T. Pirojsirikul, B. Peng, R. Peverati, J. Pittner, L. Pollack, R. M. Richard, P. Sadayappan, G. C. Schatz, W. A. Shelton, D. W. Silverstein, D. M. A. Smith, T. A. Soares, D. Song, M. Swart, H. L. Taylor, G. S. Thomas, V. Tipparaju, D. G. Truhlar, K. Tsemekhman, T. Van Voorhis, Vázquez-Mayagoitia, P. Verma, O. Villa, A. Vishnu, K. D. Vogiatzis, D. Wang, J. H. Weare, M. J. Williamson, T. L. Windus, K. Woliński, A. T. Wong, Q. Wu, C. Yang, Q. Yu, M. Zacharias, Z. Zhang, Y. Zhao and R. J. Harrison, *J. Chem. Phys.*, 2020, **152**, 184102.

29 M. M. Francl, W. J. Pietro, W. J. Hehre, J. S. Binkley, M. S. Gordon, D. J. DeFrees and J. A. Pople, *J. Chem. Phys.*, 1982, **77**, 3654–3665.

30 J. Zheng, X. Xu and D. G. Truhlar, *Theor. Chem. Acc.*, 2011, **128**, 295–305.

31 GitHub - duartegroup/otherm: thermochemical contributions from ORCA calculations, https://github.com/duartegroup/otherm, (accessed 24 December 2019).

32 S. Grimme, *Chem. - A Eur. J.*, 2012, **18**, 9955–9964.

33 H. A. Früchtl, R. A. Kendall, R. J. Harrison and K. G. Dyall, *Int. J. Quantum Chem.*, 1997, **64**, 63–69.

34 F. Weigend, *Phys. Chem. Chem. Phys.*, 2002, **4**, 4285–4291.

35 F. Weigend, *J. Comput. Chem.*, 2008, **29**, 167–175.

36 J. G. Brandenburg, C. Bannwarth, A. Hansen and S. Grimme, *J. Chem. Phys.*, 2018, **148**, 064104.

37 A. D. Becke, *J. Chem. Phys.*, 1998, **107**, 8554.

38 H. J. C. Berendsen, J. P. M. Postma, W. F. Van Gunsteren, A. Dinola and J. R. Haak, *J. Chem. Phys.*, 1984, **81**, 3684–3690.

39 G. J. Martyna, M. E. Tuckerman, D. J. Tobias and M. L. Klein, *Mol. Phys.*, 1996, **87**, 1117–1157.

40 D. D. Johnson, *Phys. Rev. B*, 1988, **38**, 12807–12813.

41 S. Tomov, J. Dongarra and M. Baboulin, *Parallel Comput.*, 2010, **36**, 232–240.

42 S. Tomov, R. Nath, H. Ltaief and J. Dongarra, in *Proc. of the IEEE IPDPS'10*, IEEE Computer Society, Atlanta, GA, 2010, pp. 1–8.

43 J. Dongarra, M. Gates, A. Haidar, J. Kurzak, P. Luszczek, S. Tomov and I. Yamazaki, *Numer. Comput. with GPUs*, 2014, 1–26.

44 R. Nath, S. Tomov and J. Dongarra, *Int. J. High Perform. Comput. Appl.*, 2010, **24**, 511–

515.

45 R. Nath, S. Tomov and J. Dongarra, in *Proceedings of the 2009 International Meeting on High Performance Computing for Computational Science, VECPAR'10*, Springer, Berkeley, CA, 2010.

46 M. Gaus, Q. Cui and M. Elstner, *J. Chem. Theory Comput.*, 2011, **7**, 931–948.

47 M. Kubillus, T. Kubař, M. Gaus, J. Řezáč and M. Elstner, *J. Chem. Theory Comput.*, 2015, **11**, 332–342.

48 C. Caleman, P. J. Van Maaren, M. Hong, J. S. Hub, L. T. Costa and D. Van Der Spoel, *J. Chem. Theory Comput.*, 2012, **8**, 61–74.

49 D. van der Spoel, P. J. van Maaren and C. Caleman, *Bioinformatics*, 2012, **28**, 752–753.

50 M. J. Abraham, T. Murtola, R. Schulz, S. Páll, J. C. Smith, B. Hess and E. Lindah, *SoftwareX*, 2015, **1**–**2**, 19–25.

51 A. Bondi, *J. Phys. Chem.*, 1964, **68**, 441–451.

52 S. S. Batsanov, *Inorg. Mater.*, 2001, **37**, 871–885.

53 M. A. Bredig, *J. Phys. Chem.*, 1942, **46**, 801–819.

54 S. Konar, J. Nylén, G. Svensson, D. Bernin, M. Edén, U. Ruschewitz and U. Häussermann, *J. Solid State Chem.*, 2016, **239**, 204–213.

55 M. Knapp and U. Ruschewitz, *Chem. - Eur. J.*, 2001, **7**, 874–880.

56 M. Atoji and R. C. Medrud, *J. Chem. Phys.*, 1959, **31**, 332–337.

57 M. Atoji, *J. Chem. Phys.*, 1971, **54**, 3514–3516.

58 A. Kulkarni, K. Doll, J. C. Schön and M. Jansen, *J. Phys. Chem. B*, 2010, **114**, 15573–15581.

59 E. Ruiz and P. Alemany, *J. Phys. Chem.*, 1995, **99**, 3114–3119.

60 J. R. Long, R. Hoffmann and H. J. Meyer, *Inorg. Chem.*, 1992, **31**, 1734–1740.

61 M. Cossi, N. Rega, G. Scalmani and V. Barone, *J. Comput. Chem.*, 2003, **24**, 669–681.

62 A. V. Marenich, C. J. Cramer and D. G. Truhlar, *J. Phys. Chem. B*, 2009, **113**, 6378–6396.
